# The Cousa objective: a long-working distance air objective for multiphoton imaging in vivo

**DOI:** 10.1038/s41592-023-02098-1

**Published:** 2023-12-21

**Authors:** Che-Hang Yu, Yiyi Yu, Liam M. Adsit, Jeremy T. Chang, Jad Barchini, Andrew H. Moberly, Hadas Benisty, Jinkyung Kim, Brent K. Young, Kathleen Heng, Deano M. Farinella, Austin Leikvoll, Rishaab Pavan, Rachel Vistein, Brandon R. Nanfito, David G. C. Hildebrand, Santiago Otero-Coronel, Alipasha Vaziri, Jeffrey L. Goldberg, Anthony J. Ricci, David Fitzpatrick, Jessica A. Cardin, Michael J. Higley, Gordon B. Smith, Prakash Kara, Kristina J. Nielsen, Ikuko T. Smith, Spencer LaVere Smith

**Affiliations:** 1 Department of Electrical and Computer Engineering, University of California Santa Barbara, Santa Barbara, CA USA; 2 Department of Molecular, Cellular, and Developmental Biology, University of California Santa Barbara, Santa Barbara, CA USA; 3 Max Planck Florida Institute for Neuroscience, Jupiter, FL USA; 4 Department of Neuroscience, Yale University, New Haven, CT USA; 5 Department of Otolaryngology, Washington University School of Medicine, St. Louis, MO USA; 6 Spencer Center for Vision Research, Byers Eye Institute, School of Medicine, Stanford University, Palo Alto, CA USA; 7 Neurosciences Interdepartmental Program, Stanford University, Stanford, CA USA; 8 Department of Neuroscience, University of Minnesota, Minneapolis, MN USA; 9 Department of Biomedical Engineering, University of Minnesota, Minneapolis, MN USA; 10 Department of Molecular and Comparative Pathobiology, and Zanvyl Krieger Mind/Brain Institute, Johns Hopkins University, Baltimore, MD USA; 11 Solomon H. Snyder Department of Neuroscience, and Zanvyl Krieger Mind/Brain Institute, Johns Hopkins University, Baltimore, MD USA; 12 Laboratory of Neural Systems, The Rockefeller University, New York, NY USA; 13 Laboratory of Neurotechnology and Biophysics, The Rockefeller University, New York, NY USA; 14 Kavli Neural Systems Institute, The Rockefeller University, New York, NY USA; 15 Department of Otolaryngology, Stanford University School of Medicine, Stanford University, Stanford, CA USA; 16 Department of Molecular and Cellular Physiology, Stanford University School of Medicine, Stanford University, Stanford, CA USA; 17 Department of Psychology and Brain Sciences, University of California Santa Barbara, Santa Barbara, CA USA; 18 Neuroscience Research Institute, University of California Santa Barbara, Santa Barbara, CA USA

**Keywords:** Neuroscience, Multiphoton microscopy

## Abstract

Multiphoton microscopy can resolve fluorescent structures and dynamics deep in scattering tissue and has transformed neural imaging, but applying this technique in vivo can be limited by the mechanical and optical constraints of conventional objectives. Short working distance objectives can collide with compact surgical windows or other instrumentation and preclude imaging. Here we present an ultra-long working distance (20 mm) air objective called the Cousa objective. It is optimized for performance across multiphoton imaging wavelengths, offers a more than 4 mm^2^ field of view with submicrometer lateral resolution and is compatible with commonly used multiphoton imaging systems. A novel mechanical design, wider than typical microscope objectives, enabled this combination of specifications. We share the full optical prescription, and report performance including in vivo two-photon and three-photon imaging in an array of species and preparations, including nonhuman primates. The Cousa objective can enable a range of experiments in neuroscience and beyond.

## Abstract (web summary)

The Cousa objective is an ultra-long working distance air objective optimized for two- and three-photon imaging. Bypassing challenges caused by water immersion and short working distances, the Cousa enables and improves imaging of diverse specimens.

## Main

Multiphoton microscopy of in vivo neuronal activity has been transformative for neuroscience, but its application can be complicated due to the limitations of microscope objectives^1–6^. Conventional microscope objectives with good multiphoton performance often have short working distances (WDs) (1–10 mm) and/or require water immersion. The short working distance, coupled with the geometry of the objective tip, can complicate imaging in larger mammals^2,7^, requiring excessive tissue removal and large cranial windows, which can exacerbate immune responses and degrade tissue clarity^8^, ultimately limiting the imaging depth and the duration of longitudinal imaging. Exotic applications such as imaging through post-objective optics^9^ including prisms^10,11^ or in complex surgical preparations can also be hampered by short WDs. Moreover, water immersion can require awkward reservoirs or the use of gels that can lack appropriate refractive indices and harbor air bubbles that degrade image quality.

To address these issues, we designed an ultra-long WD air immersion objective with an unconventional mechanical shape. It has a WD of 20 mm, an effective focal length of 20 mm (that is, a ×10 magnification) for a field of view (FOV) of more than 4 mm^2^, a numerical aperture (NA) of 0.50 and the optics are designed to minimize aberrations for two-photon imaging across a range of imaging configurations. This objective, referred to as the Cousa, was designed to be compatible with commercial multiphoton imaging systems, with standard threading and an entrance pupil of Ø20 mm. After designing, manufacturing and characterizing the optical performance, the Cousa was compared to a conventional short WD objective and the data quality was similar across the two. Next, the Cousa objective was tested in a range of experiments in various species, including mice, ferrets, tree shrews, monkeys and pigs, using commercial, off-the-shelf imaging systems. To the best of our knowledge, the Cousa enabled the first two-photon calcium imaging in cochlear hair cells in vivo, the first two-photon imaging of porcine retina through the intact eye, and longest WD large FOV three-photon and third-harmonic generation (THG) imaging in vivo.

## Results

### Design

#### Specifications and constraints

**Fig. 1:**
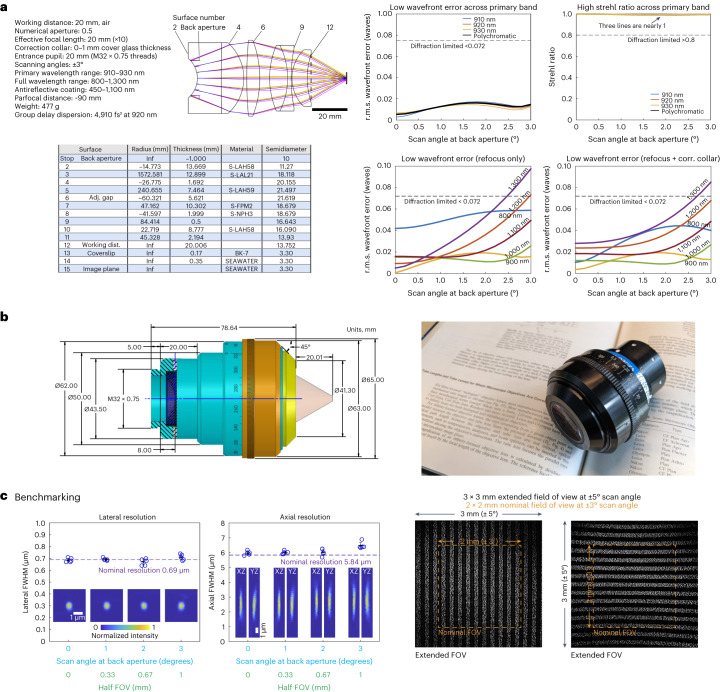
Design and benchmarking. **a**, Left: the specifications that constrained the design to ensure compatibility with two-photon imaging systems that are typically used in vivo. The resulting optical design has six elements and one adjustable air gap (adjustment range 5.4–6.0 mm) to optimize performance. The full lens prescription is provided. Right: the primary optimizations were for 920 ± 10 nm for two-photon excitation of GFP-based indicators. The optical model predicts low r.m.s. wavefront errors and high Strehl ratios for 910, 920 and 930 nm light across the scan angles of 0–3°, well beyond the diffraction limit. Performance is also diffraction-limited across a broader wavelength range from 800 to 1,300 nm. The r.m.s. wavefront error remains below the diffraction limit for most of the 0–3° scan angle range, when the focal plane is allowed to naturally shift with wavelength, and the correction (corr.) collar provides an additional degree of optimization. **b**, Left: the mechanical design of the objective prioritized keeping the widest diameter near the middle of the objective to avoid mechanical collisions with objective mounts. All dimensions are in mm unless otherwise noted. Right: a photograph of the manufactured objective. **c**, Left: two-photon excitation PSF measurements were made with 0.2 µm beads embedded in agar at a depth of 350 µm. The excitation wavelength is 910 nm. *z* stack images are acquired for beads at four lateral locations including on axis, 1°, 2° and 3° off axis (*n* = 5 beads at each location). FWHM of the Gaussian fits for measurements (mean values ± s.d.) indicate lateral and axial resolutions indistinguishable from diffraction-limited resolutions. The pixel size of the images is 0.058 × 0.064 × 0.69 µm^3^ (*xyz*). Right: images of a fluorescent calibration sample with a periodic line pattern (five lines per millimeter) in two orientations acquired under a ±5° scan angle show a nominal 2 × 2 mm^2^ FOV of the objective under the ±3° scan angle, and a 3 × 3 mm^2^ FOV under ±5° scan angle.

The design specifications of the Cousa objective (Fig. 1a) were set and balanced primarily around three factors: (1) geometric parameters to facilitate use in animal imaging applications, (2) optimization for two-photon imaging across a large FOV with subcellular resolving power and (3) compatibility with commercial two-photon imaging systems.

First, its raison d’être, is compatibility with animal experiments and the associated instrumentation. Two-photon imaging in neuroscience is often performed through a cranial window, with the objective above the window, at a distance determined by WD, which can pose constraints on imaging. For example, ferrets and other animals have skulls that are more than 1 mm thick with a large gap between the skull and dura mater. In these cases, cranial windows must be enlarged to accommodate standard two-photon objectives due to their short WDs and tip geometries. However, large imaging windows create challenges for window positioning, imaging quality and long-term maintenance. Even in smaller animals such as mice, short WDs prevent the insertion of auxiliary optics between the objective and sample, and can also prevent imaging in complex preparations. To address these issues, we started with the requirement that the WD would be long: 20 mm. A long WD design allows the objective to remain comfortably outside an imaging chamber, resulting in fewer mechanical constraints. We also recognized that imaging at angles other than the conventional vertical orientation can make maintaining consistent, bubble-free water immersion difficult. Thus, we chose to use air immersion. Air immersion entails a larger refractive index mismatch than water immersion designs, so we mitigated the trade-off by incorporating a correction collar that can compensate for aberrations (Fig. 1a).

Second, the lens design was optimized for focusing ultrafast laser pulses centered at wavelengths commonly used in two-photon imaging, including popular genetically encoded calcium indicators such as the GCaMP series^12–14^. The optics were designed to offer diffraction-limited performance across a range of wavelengths. We set the NA to be 0.50 (Fig. 1a), corresponding to a diffraction-limited resolution of 0.69 µm laterally and 5.84 µm axially, which is sufficient to resolve neurons, dendritic spines and axonal boutons^15,16^.

Third, the objective was designed to be compatible with commercial multiphoton imaging systems. Major microscope manufacturers use infinity-conjugate (that is, infinity-corrected) optical designs^17^, and thus we adopted the same convention for compatibility. The other constraints from commercial systems are the beam diameter at the objective back aperture and the maximal scan angle. These two parameters are determined by the scan engine. Many commercial systems constrain the maximal beam diameter to roughly 20 mm, and the scan angles to roughly ±3°. A popular short WD water immersion objective, the Nikon ×16/0.8 NA (CFI75 LWD 16X W) has a back aperture of 20 mm and is often used with a variety of commercial systems. With these specifications and constraints set (WD, air immersion, NA, scan angle and back aperture diameter), a relatively large FOV remains feasible by setting the effective focal length of the objective to 20 mm (ref. ^18^). The design and optimization process was conducted using optical simulation software (Zemax OpticStudio). The merit function prioritized maximizing the WD while maintaining a diffraction-limited point spread function (PSF) and minimizing the wavefront error across the FOV. Parallel efforts were made to reduce the number of lenses and the thickness of the materials, and thus minimize the size, weight and cost of the final design.

#### Design and model performance

**Extended Data Fig. 1:**
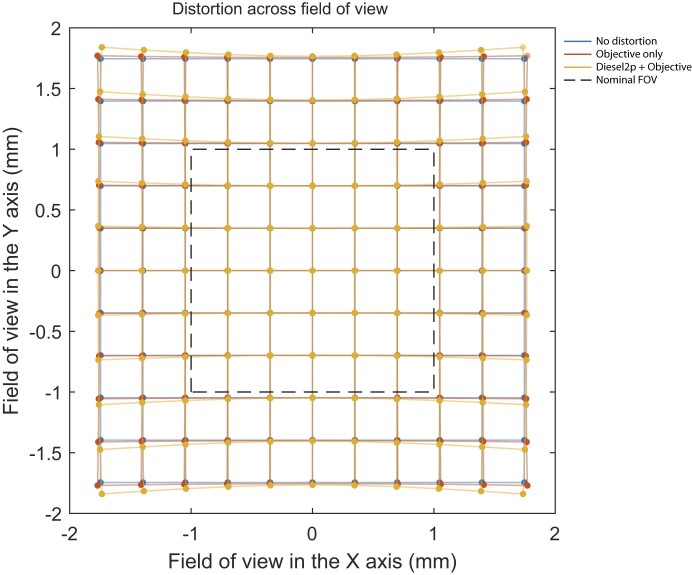
Field distortion. The diagram shows the distortion of the field of view simulated from the Zemax model. The blue grid shows the undistorted (square) FOV for comparison. The orange grid shows the shape of the FOV for the Cousa objective, showing a pincushion distortion. The yellow grid shows the shape of the FOV formed by the sum of the Diesel2p scan engine and the Cousa objective, showing a combination of the pincushion and barrel distortion. The shape of the simulated distortion (in yellow) matches with the distortion of the experimental measurement shown in Fig. 1c. Note that there is nearly zero field distortion in the 2 × 2 mm^2^ nominal FOV (dashed black square).

**Extended Data Fig. 2:**
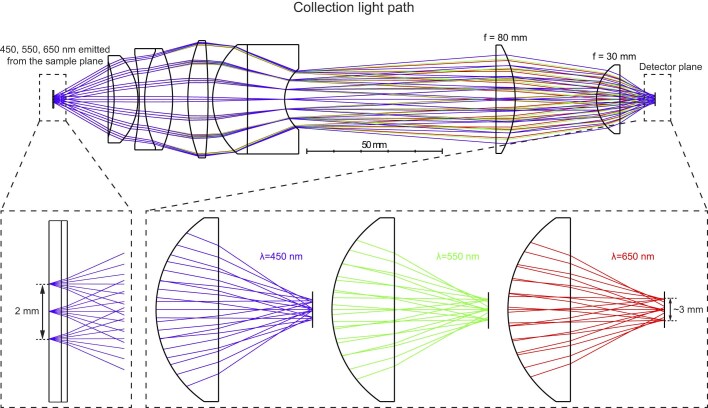
Collection light path. A schematic shows the ray-traces in the collection light path for the Cousa objective. Fluorescence photons with wavelengths in the visible band (450 nm, 550 nm, and 650 nm) are emitted from the imaging plane within the 2 mm nominal FOV (zoom in view on the left), passing through the Cousa objective. These photons are further collected and guided by the optical relay, and reach at the cathode of the photomultiplier (PMT). The optical relay is constructed simply with two off-the-shelf singlet lenses from OptoSigma (011-2570-a55) and Thorlabs (LA1805-A). The zoom in view in the bottom-right shows that all of the emitted photons (rays) in the visible spectrum reach at the detector surface within a 3 mm wide spread.

The infinity-corrected objective consists of six lens elements (Fig. 1a) with a net group delay dispersion of roughly 4,910 fs^2^ at 920 nm (ref. ^19^). The WD (surface 12 to the focal plane at surface 15) is roughly 20 mm. The position of the back aperture was designed to be very close to the first element, surface 2. This facilitates alignment in commercial systems, since visual inspection at the back surface can determine whether the excitation beam remains stationary during scanning. Distortions were also minimized across the FOV (Extended Data Fig. 1). Although achromatic performance across visual wavelengths was not a priority (as it commonly is with many conventional wide-field and confocal objectives), we also ensured that the collected fluorescence over the visible spectrum of 450–650 nm was focused to a small area (roughly 3 mm) for compatibility with popular photodetectors (Extended Data Fig. 2).

**Extended Data Fig. 3:**
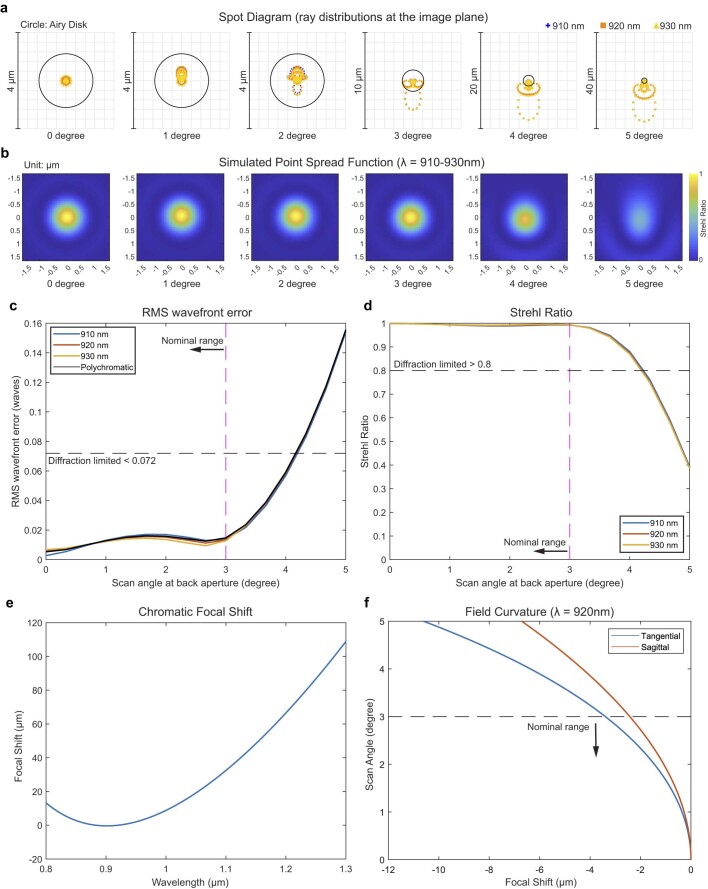
Additional information on performance. Additional information on nominal performance. **(a)** The spot diagram shows the distribution of rays at the imaging plane coming from a scan angle of 0°, 1°, 2°, 3°, 4°, 5°, aiming at the objective’s back aperture with a beam diameter of 20 mm, and with wavelengths of 910 nm, 920 nm, and 930 nm. **(b)** The simulated point spread functions from Zemax at the 5 scan angles are shown. The root-mean-square wavefront error **(c)** and the Strehl ration **(d)** as a function the scan scan angle at the back aperture is shown for the wavelength of 910 nm, 920 nm, 930 nm, and all three combined (polychromatic). The horizontal dashed line indicates that the curve below 0.072 in the RMS wavefront error plot and above 0.8 in the Strehl plot is diffraction limited. The vertical dashed line in (c) and (d) shows the nominal range of scan angle is smaller than 3 degrees. **(e)** The dependence of focal shift on the wavelength in the center of FOV is shown. **(f)** The depth of the tangential and sagittal focus over a scan angle of 5 degrees at 920 nm is shown. The horizontal dashed line shows the nominal range of the scan angle < 3°. There is only 4 µm depth difference in the center (0°) and in the edge (3°) of the FOV within the nominal scan range.

The root-mean-square (r.m.s.) wavefront error for 920 ± 10 nm light is less than 0.02*λ* across the scan angles, which is considerably less than the Maréchal criterion of 0.072*λ* (lower is better) (Fig. 1a), which is a diffraction limit criterion. Similarly, the Strehl ratio^20^ is more than 0.97 across the nominal ±3° scan angles (Fig. 1a), exceeding the diffraction limit of 0.8 (higher is better). This performance is maintained across and beyond the nominal FOV (Extended Data Fig. 3). Thus, the performance is diffraction-limited throughout the designed FOV by a large margin, which provides some assurance that performance will remain diffraction limited despite real-world imperfections that are incorporated during fabrication and assembly.

**Extended Data Fig. 4:**
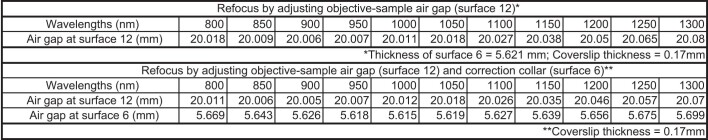
Thickness of air gaps for optimal performance at different wavelengths. The nominal thicknesses of air gaps used for different wavelengths. The upper portion of table lists the distance of the objective-to-sample gap (Surface 12) when this single air gap is adjusted for different wavelengths. The lower part of the table shows the thicknesses of the objective-to-sample gap (Surface 12) and the correction collar gap (Surface 6) when both of these two air gaps are adjusted.

**Extended Data Fig. 5:**
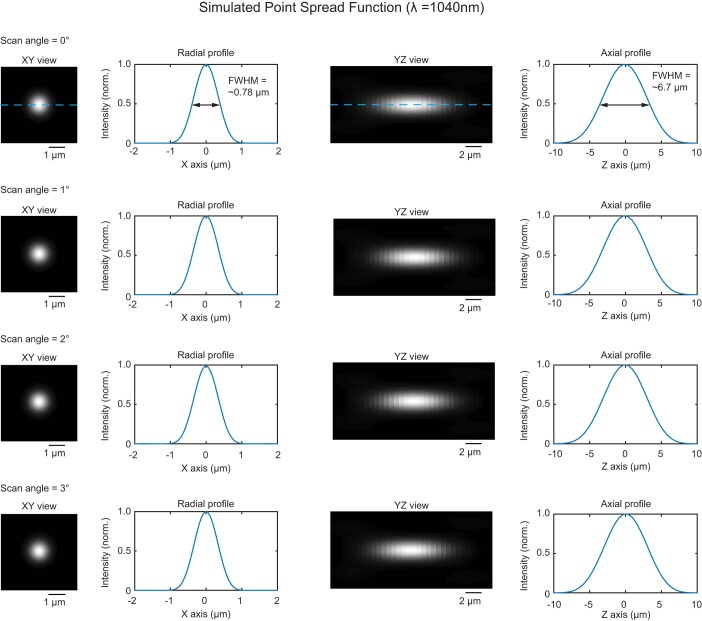
Focusing 1040 nm light. Across scan angles from 0 to 3 degrees, the focus of 1040 nm light is < 0.8 µm laterally and < 7.0 µm axially.

**Extended Data Fig. 6:**
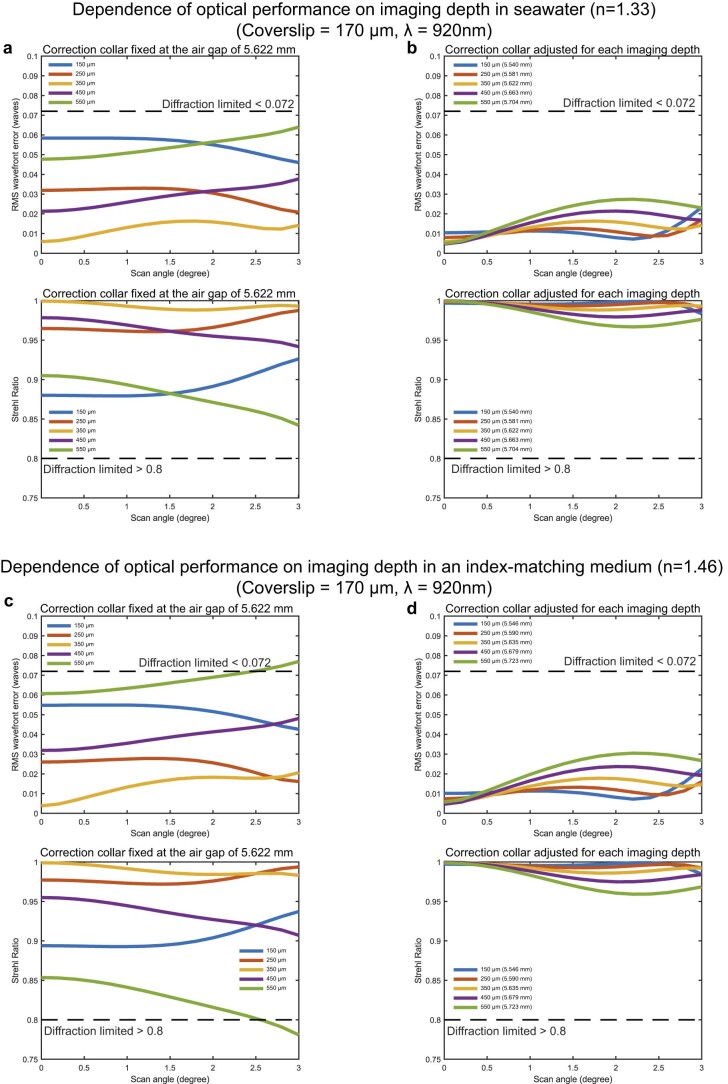
Dependence of performance on the imaging depth and refractive index of the sample medium as well as the compensation using the correction collar. The root-mean-squared (RMS) wavefront error (top) and the Strehl ratio (bottom) as a function of the scan angle at the back aperture at different imaging depths inside seawater (n = 1.33) are shown with the correction collar fixed at an air gap of 5.622 mm in **(a)**, and with the correction collar optimized for different imaging depths in **(b)**. Different imaging depths ranging from 150-550 µm are color coded. The same plots inside an index-matching medium (n = 1.46) are shown as (a) with the correction collar fixed at an air gap of 5.622 mm in **(c)**, and with the correction collar optimized for different imaging depths in **(d)**.

**Extended Data Fig. 7:**
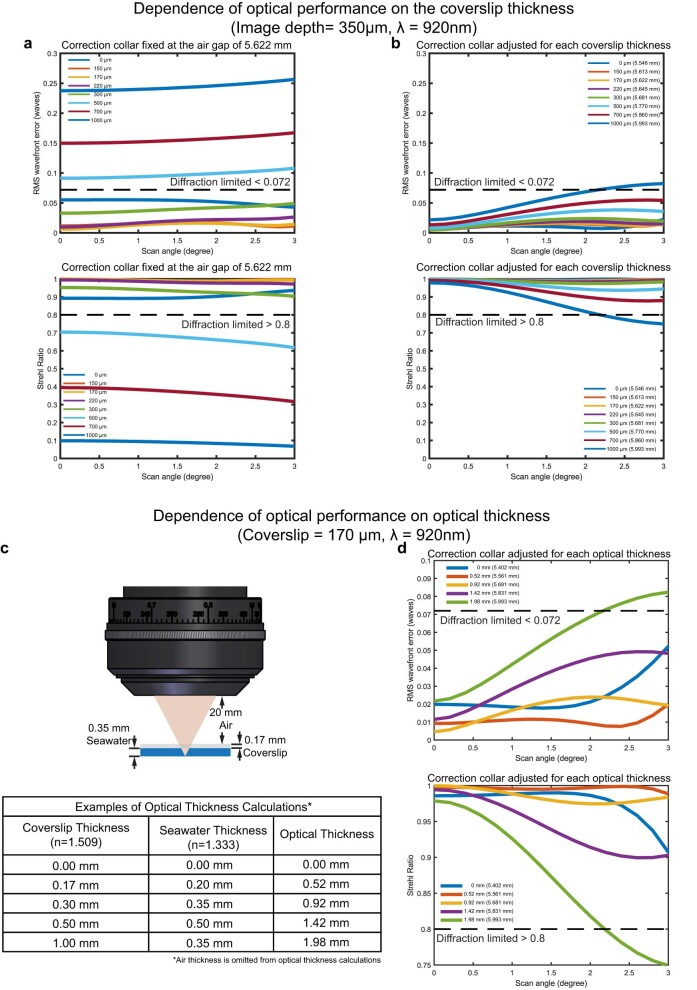
Dependence of performance on coverslips of different thickness and the compensation using the correction collar. The root-mean-squared (RMS) wavefront error (top) and the Strehl ratio (bottom) as a function of the scan angle at the back aperture and coverslips of different thickness are shown with the correction collar fixed at an air gap of 5.622 mm in **(a)**, and with the correction collar optimized for coverslips of different thickness in **(b)**. The thickness of coverslips ranging from 0-1000 µm are color coded. **(c)** The schematic shows the Cousa objective focuses light through a 0.17 mm thick coverslip and 0.35 mm of seawater. The optical thickness of the non-air elements between the objective and the focal plane can be calculated: multiply the thickness of a material (ignoring air) in millimeters by its refractive index, and sum over all of these products. For example, the optical thickness shown in the schematic is 0.723 (0.17 mm × 1.509 + 0.35 mm × 1.333). The table below shows combinations of the coverslip thickness and the seawater thickness, and the resultant optical thickness for each combination. The correction collar can be adjusted to compensate for the optical thickness over the range of 0 - 2 mm, and maintain the diffraction-limited performance. **(d)** The root-mean-squared (RMS) wavefront error (top) and the Strehl ratio (bottom) as a function of the scan angle at the back aperture are shown when the correction collar is optimized for different optical thickness of non-air materials. Each curve is color coded for different optical thickness shown as the number before the parenthesis. The number in the parenthesis is the optimal thickness of air gap to achieve the best optical performance.

The objective has a tunable air gap between surfaces 6 and 7 (adjustment range 5.4–6.0 mm) that is adjusted by a rotating correction collar. Correction collar adjustments can compensate for a range of cover glass (surface 13) thicknesses, from 0 to 1.0 mm. The correction collar can also be adjusted to optimize performance at different excitation wavelengths (Fig. 1a). By adjusting the correction collar position and permitting the movement of the focal plane, diffraction-limited performance can be extended to a range of 800–1,300 nm, which covers the range of commonly used multiphoton excitation wavelengths (Fig. 1a and Extended Data Figs. 4 and 5). This adjustment can also optimize performance for a range of imaging depths (Extended Data Fig. 6a,b) or samples of varying refractive indices (Extended Data Fig. 6c,d) and coverslips of varying thicknesses (Extended Data Fig. 7). Note that the refocusing is applied at a single position for all scan angles, and the merit function balances performance over the full FOV. Thus, the correction collar enables users to optimize performance over a range of experimental parameters.

#### Mechanical model and assembly

After optical designs were finalized, the mechanical design, lens fabrication, housing manufacturing and objective assembly processes were contracted to an external firm (Special Optics). The objective is 79 mm long and 65 mm wide at its widest point. Its total weight is 477 g (Fig. 1a). The long WD of the objective relaxes the geometric constraints of the design, as was our strategy. However, one mechanical constraint remained: the objective needed to fit within the clearance around the objective mounting threads of commonly used multiphoton microscopes. A conventional way to load the optics into an objective is to leave the back open, insert all lenses and then seal it off. This stacking approach leads to the largest diameters being at the back of the objective near the threads. Realizing this problem in an early version, we redesigned the optomechanics for assembly in the middle at the adjustable air gap surface (surface 6). Lenses are loaded from this plane into both halves and then the two halves are joined. This reduces the diameter of the shoulder near the threads, and moves the largest diameter to the middle of the lens where it can be more easily accommodated on commercial multiphoton microscopes. The resulting silhouette of the objective resembles a cousa squash, and inspired the name of the objective (Fig. 1b). The total traveling range of the adjustable air gap is 1.0 mm, corresponding to 1.3 revolutions of the correction collar, with a precision of 2.08 µm per degree. The correction collar is marked to indicate both 360° around the objective and various cover glass thicknesses. The tip of the objective is beveled at 45° to gain some clearance near the sample space.

### Characterization and performance

#### Resolution, FOV and light transmission

We characterized the performance of the objective using a custom two-photon scan engine with a 32 mm diameter beam scanned over a ±5° range^15^. These scan parameters exceed the requirements of the objective (20 mm and ±3°, respectively), thus the performance should be objective limited, rather than scan engine limited^21^. We first measured the resolution attained by the objective by taking *z* stacks of 0.2 µm fluorescent beads at various positions across the FOV (Fig. 1c). The lateral full-width at half-maximum (FWHM) is roughly 0.69 µm throughout the FOV (0.69 ± 0.02 at 0°, 0.69 ± 0.01 at 1°, 0.67 ± 0.03 at 2°, 0.71 ± 0.05 at 3°, mean ± s.d., *n* = 5 at each angle), which is consistent with the theoretical diffraction-limited resolution^22^. The axial resolution is roughly 5.84 µm, again providing a good match to the theoretical value, up to ±2° scan angles and deviates by about 10% at 3° of scan angle (5.97 ± 0.13 at 0°, 6.00 ± 0.10 at 1°, 6.03 ± 0.21 at 2°, 6.47 ± 0.21 at 3°, mean ± s.d., *n* = 5 at each angle). The match between the experimental measurement and the theoretical calculation confirms that the NA of the objective is 0.50, as designed (Methods). This result also indicates that the r.m.s. wavefront error is low. Like all other infinity-corrected microscope objectives, underfilling the objective’s back aperture will lead to lower spatial resolution, so overfilling the objective is suggested to use the full excitation NA of the Cousa objective^21^.

We next measured the imaging FOV with a structured fluorescent sample with periodic lines (five per mm; item 57–905, Edmund Optics). When the scan angle is ±3°, the images contain ten lines along both the *x* and *y* directions without vignetting, indicating a 2 mm length on each axis of the FOV (Fig. 1c). The result demonstrates that the objective has a FOV of 2 × 2 mm^2^ area, consistent with the nominal model performance (Fig. 1c). The FOV can be extended to roughly 3 × 3 mm^2^ with a scan of ±5°, and vignetting occurs at the corners of the field (Fig. 1c). The distortion is due to a deviation from the F-theta condition in the scanning system of the Diesel2p (Extended Data Fig. 1)^15^. Together, the resolution and FOV provide a space-bandwidth product of (2,000 µm/0.69 µm)^2^ = 8.4 megapixels with ±3° scanning.

#### Categorical and quantitative comparisons

**Fig. 2:**
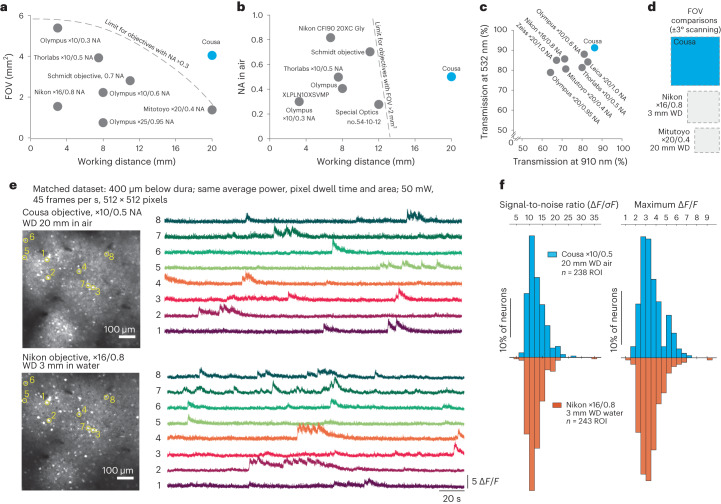
Categorical and quantitative comparisons. **a**, Conventional objectives are constrained to a mechanical envelope that limits the product of FOV and WD. The general limits imposed by this mechanical envelope are sketched with dotted lines^43^. The Cousa objective is distinctive in that it combines an ultra-long WD of 20 mm with a large FOV and an NA of 0.5. **b**, Conventional objectives that can offer a FOV >2 mm^2^ are typically constrained to shorter WD. The Cousa has a long WD, and provides more than 4 mm^2^ FOV. **c**, Overall throughput in both near IR (910 nm) and visible (532 nm) wavelengths is higher with the Cousa than conventional objectives. This is potentially due to a lower number of lenses in the Cousa. **d**, The FOV of the Cousa dwarfs both a commonly used short WD low magnification multiphoton objective (Nikon ×16/0.8 NA) and a conventional long WD objective (Mitutoyo ×20/0.4 NA). **e**, In vivo calcium dynamics (GCaMP6s) are measured with the Cousa objective and a popular water immersion objective (Nikon ×16/0.8 NA) and compared. The exact same imaging parameters are used to image the same field of neurons in the same mouse (awake, spontaneous activity). Averaged images for both objectives are shown on the left. Calcium traces from the identical ROI are shown on the right (raw data at 45 frames per s; no filtering). The data quality of the Cousa objective is similar to that of this commonly used short WD objective (Nikon ×16/0.8 NA). **f**, The signal-to-noise ratio and maximum Δ*F*/*F* of the calcium dynamic for each ROI in **e** are calculated and plotted as histograms for both objectives. The signal-to-noise ratio for each ROI is the ratio of the maximal magnitude of the calcium trace to the standard deviation of the fluctuating signal around the baseline. The maximum Δ*F*/*F* for each ROI is the maximal value of Δ*F*/*F* throughout the trace. Thus, the Cousa provides an ultra-long WD in air, a large FOV and raw data similar to those from conventional objectives.

**Extended Data Fig. 8:**
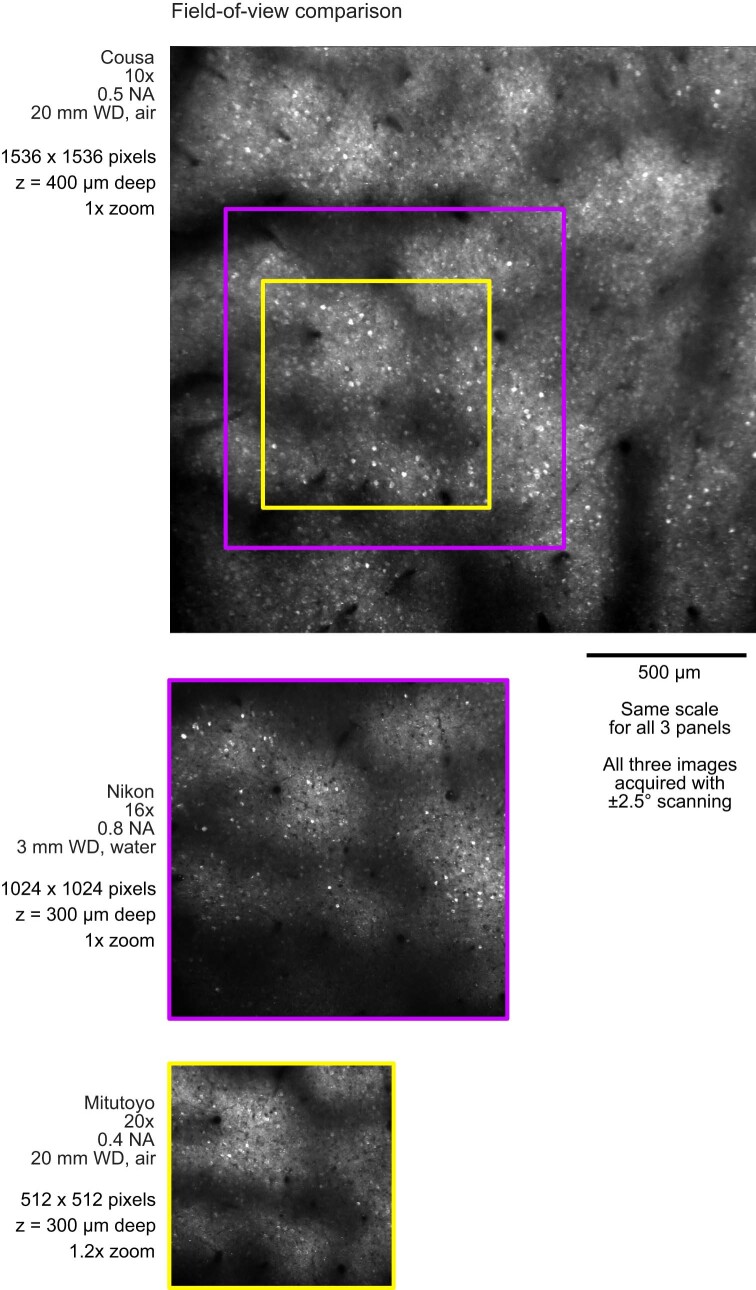
Comparing the field-of-view (FOV) between the Cousa and two conventional objectives.

This objective design is unique in its combination of parameters, which is enabled by being free from the parfocal lengths and mechanical envelopes of conventional objectives. Free from those design constraints, we could better optimize for the application. Conventional objectives with NA > 0.3 have a trade-off between WD and FOV, and objectives with a FOV > 2 mm^2^ are typically constrained to WDs shorter than 14 mm. The Cousa breaks free from these conventional limits (Fig. 2a,b). Effective antireflective coatings and a limited number of lenses ensured high light throughput. We found that 86% of 910 nm and 91% of 532 nm light were transmitted through the objective, comparing well to commonly used multiphoton imaging objectives (Fig. 2c). The FOV of the Cousa extends past its nominal 2 × 2 mm^2^ at ±3° scanning. However, we can use this specification for comparison. At those same scan angles, a commonly used short WD water immersion objective (Nikon ×16/0.8 NA) provides smaller FOV. A conventional objective that offers a 20 mm WD in air (Mitutoyo ×20/0.4 NA) is limited to an even smaller FOV (Fig. 2d and Extended Data Fig. 8).

**Extended Data Fig. 9:**
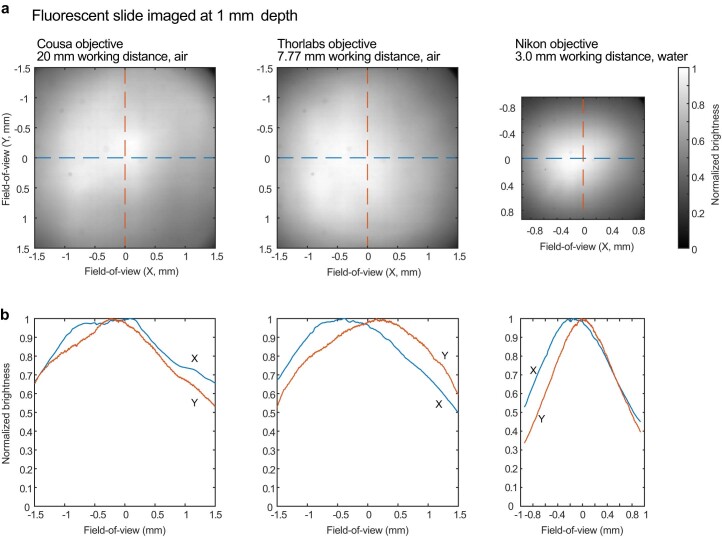
Field uniformity compared among three objectives. **(a)** Images of a fluorescent slide at 1 mm depth using the Cousa, the Thorlabs 10x/0.5NA, and the Nikon 16x/0.8NA objectives with the laser beam scanning ±5° at the back aperture. The brightness of each image was normalized to the peak brightness, individually. Scan angles of ±5° correspond to a ~ 3 mm-wide field-of-view for the Cousa and Thorlabs 10x objectives, and a ~ 1.8 mm-wide field-of-view for the Nikon 16x objective. **(b)** The profiles of normalized brightness along the colored, dashed lines in (a). Note that the Cousa and the Thorlabs 10x/0.5NA have similar field uniformity, despite the Cousa being >12 mm further from the focal plane. The field brightness drops off rapidly from the center for the Nikon 16x/0.8NA objective.

While the 20 mm WD and large FOV are categorically new to neuroscience, we sought to compare signal quality from the use of the Cousa to a popular short WD water immersion objective with a higher NA (Nikon ×16/0.8 NA). When using the same average power (50 mW after the objective), the same pixel dwell times, FOV and pixel counts, the calcium signals measured in vivo at a depth of 400 µm were difficult to distinguish between the two objectives (Fig. 2e,f), thus providing confidence that high fidelity data can be obtained with the Cousa. The similarity of the data between the two objectives was likely due to several factors. On the excitation side, the Nikon ×16/0.8 NA objective probably did not achieve the full NA due to the loss of marginal rays in the tissue, while the lower NA Cousa may have experienced less loss of NA^23,24^. On the detection side, the longer focal length of the Cousa, and thus a larger FOV to collect scattered photons, may have partially offset its lower NA. Although the same average power was used out of the objective, on the collection side, the slightly higher transmission of the Cousa would also help make up the difference. To compare the field uniformity among different objectives, we imaged a fluorescent slide at 1 mm depth and ±5° scanning with the Cousa, the Thorlabs ×10/0.5 NA and the Nikon ×16/0.8 NA objectives (Extended Data Fig. 9). The Cousa and the Thorlabs ×10/0.5 NA have similar field uniformities, despite the fact that the Cousa objective is more than 12 mm further from the sample (20 mm versus 7.8 mm WD). In addition, the field uniformity drops off more quickly at the edges of the ±5° FOV with the Nikon ×16 objective than with the Cousa objective. Overall, the Cousa provides high fidelity data in vivo, 400 µm deep into densely labeled tissue, which compares well with a popular short WD objective (Fig. 2e,f).

#### In vivo two-photon calcium imaging of mouse neural circuitry

**Fig. 3:**
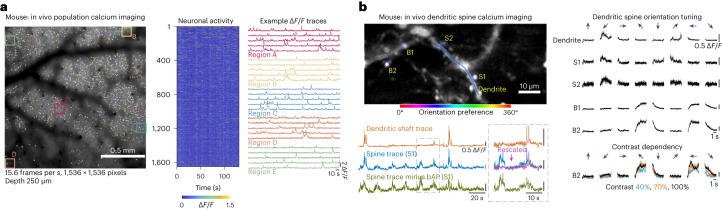
Two-photon calcium imaging of soma, dendrites, spines and boutons. **a**, Population calcium imaging (GCaMP6s) over a 1.7-mm-wide FOV. Traces from cells within boxes at left are expanded at right, a selection of the 1,648 neurons detected in this dataset. **b**, In a mouse with ultra-sparse expression of GCaMP8m in V1, we imaged calcium transients in putative axonal boutons (B), dendritic spines (S) and dendritic shafts during the presentation of visual stimuli (drifting gratings). Color codes show the orientation preference of each ROI. bAP-associated calcium transients detected in the dendrite were subtracted from the dendritic spine S1 signal, revealing activity events that are independent from local bAP signals, indicative of local synaptic input. Orientation tuned responses were reliable for spines S1 and S2, boutons B1 and B2, and the nearby dendrite (*n* = 15 repeats per stimulus; mean in black ± s.e.m. in gray). Responses in axonal bouton B2 varied with contrast (contrast levels of 40% in blue, 70% in orange and 100% in black; mean ± s.e.m.; *n* = 5 repeats).

After benchmarking and validating the optical performance of the objective, the Cousa objective was used on a range of multiphoton imaging systems, including custom-built systems and commercial systems from Bruker, Thorlabs, Neurolabware and Sutter. These systems vary in their scan engine performance (for example, beam size can vary and affect resolution^21^), and thus can provide examples of real-world performance of the objective in experiments. As a first test, a cranial window was implanted in a transgenic mouse with neurons expressing the genetically encoded calcium indicator GCaMP6s^25^. The Cousa objective was mounted on a custom microscope that provided a ±2.6° scan angle range and a 20 mm diameter beam at the back aperture of the objective. This system also had a 12 kHz resonant scanner for fast raster scanning. First, a *z* stack image series was acquired covering the volume of 1 × 1 × 0.5 mm^3^ (*xyz*) (Supplementary Video 1). These data demonstrated that individual neurons were resolved up to the depth of 0.5 mm. Next, a FOV of 1.7 × 1.7 mm^2^ was recorded using the full ±2.6° scan angle at back aperture (Fig. 3a and Supplementary Video 2) with 1,536 scan lines, 1,536 pixels per scan line and a frame rate of 15.4 frames per second. Spontaneous calcium transients at a imaging depth of 250 µm were imaged from 1,648 neurons detected throughout the FOV (Fig. 3a). Calcium indicator traces from neurons across the FOV exhibited high Δ*F*/*F* signals (Fig. 3a). These in vivo results demonstrate performance in the target application, with a relatively large FOV, even when using relatively short pixel dwell times (roughly 28 ns per pixel or roughly two pulses per pixel).

We next performed two-photon imaging of dendrites and axons in a mouse that sparsely expressed GCaMP8m. Neuronal activity in the primary visual cortex (V1) was imaged while the animal viewed black and white drifting gratings of eight different orientations (0–315° and 45° steps). The spines and their local dendritic shaft are clearly resolved, and some putative boutons are identified with distinctive calcium activity (Fig. 3b). Dendritic spine transients showed clear independent calcium dynamics in addition to those associated with back-propagating action potentials (bAP), demonstrating that the fluorescence signals from the spine and its parent dendrites can be unambiguously extracted. Together, the bAP signals can be removed from the spine with high fidelity (Fig. 3b)^12^. These identified spines, boutons and dendrites show reliable responses to visual stimuli and vary in terms of which stimulus orientations elicit the strongest responses (Fig. 3b). Moreover, response magnitude of the axonal bouton transients showed contrast-dependence (40, 70 and 100%), further highlighting the sensitivity of the objective and performance in a challenging experiment (Fig. 3b). Taken together, these results demonstrate that the Cousa objective has not only high resolution for resolving synapses and small neuronal processes, but also sufficient two-photon excitation and collection efficiency to detect fine changes in calcium transients.

**Fig. 4:**
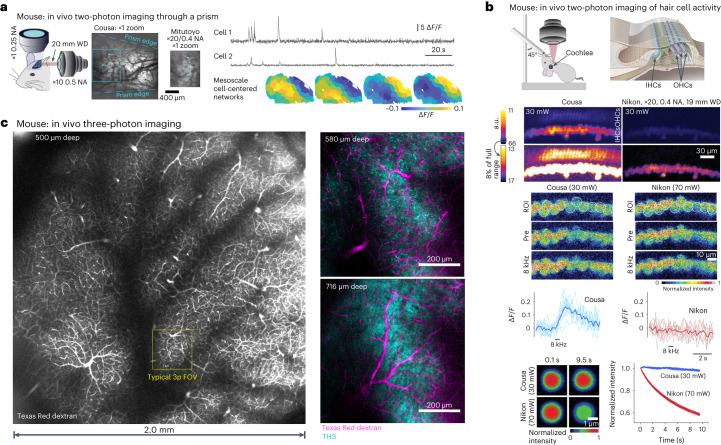
Two-photon and three-photon imaging in mice. **a**, Simultaneous two-photon imaging (through a prism) and mesoscopic wide-field imaging in awake, head-fixed mice obtained a larger FOV with the Cousa. Time-averaged two-photon image obtained through the prism show the difference in FOV compared with a commercially available 20 mm air objective. Time series for neurons imaged with two-photon excitation through the Cousa objective and microprism were used to detect cell-centered networks for 74 neurons in one mouse. **b**, Top: the Cousa objective enables in vivo functional imaging of cochlear hair cells. The mouse is held supine for imaging of IHCs and OHCs. We imaged with both the Cousa objective and a conventional objective, the Nikon ×20/0.4 NA, 19 mm WD (TU Plan ELWD 20X), with the same laser power (30 mW). Middle: for calcium imaging in IHCs, the Cousa was used with 30 mW and the Nikon was used with 70 mW, to obtain minimal usable signal levels for both at 5.7 frames per s. In response to sound stimulation, IHCs exhibited responses only during Cousa imaging. Bottom: fluorescent particles (diameter 5.63 µm) faded rapidly when imaged with the Nikon, and maintained fluorescence when imaged with the Cousa. a.u., arbitrary units. **c**, Left: the Cousa supports large FOV three-photon imaging, with a 20 mm WD. The vasculature across the entire 4 mm^2^ region is visible after an intravenous injection of Texas Red dextran. Right: higher zoom single *z* plane three-photon images from a second mouse with dual channel imaging of Texas Red dextran (magenta) and THG (cyan) in cortex and white matter.

Short WD objectives preclude the implementation of intermediate optics between the objective and the sample, such as prisms, mirrors and gradient refractive index lenses^9–11,26,27^. Long WD air objectives can enable experiments such as simultaneous mesoscopic and two-photon imaging of neuronal activity using a prism in the post-objective space^10^. In this method, dual asymmetric imaging pathways are used to record the activity of individual neurons relative to ongoing, large-scale dynamics across the dorsal neocortex. The Cousa objective was mounted horizontally and used in conjunction with a microprism implanted on the cortical surface (Fig. 4a)^10^. Compared to previously used instrumentation (Mitutoyo ×20/0.4 NA), the Cousa objective offered a larger FOV, enabling the simultaneous imaging of a larger population of neurons for correlation analysis.

#### In vivo calcium imaging of the mouse cochlea

The peripheral hearing organ, the cochlea, is difficult to reach surgically and optically. Moreover, it is a mechanosensitive, fluid-filled structure, which further complicates surgical preparations and functional imaging^28,29^. A long WD objective is required to keep the neighboring hearing structures intact (Fig. 4b), and air immersion is needed to preserve sound transference through the air-filled middle ear cavity in vivo. In this application, we directly compared the performance of the Cousa objective with a conventional objective (TU Plan ELWD ×20, 0.4 NA, 19 mm WD; Nikon), which was formerly a leading objective for this preparation^28^. In vivo two-photon cochlear images were first collected with the same laser power (30 mW) in a genetically modified mouse expressing tdTomato in hair cells. The signal intensity from inner and outer hair cells (IHCs and OHCs) when using the Cousa objective was higher than that from the Nikon objective (Fig. 4b). Next, a mouse expressing GCaMP6s selectively in HCs was used to functionally monitor sound-evoked Ca^2+^ responses in the cochlea in vivo (Fig. 4b). An 8 kHz pure tone was played for 0.5 s to stimulate the IHCs at the imaging location. While 30 mW laser power was enough to resolve the Ca^2+^ sensor in IHCs with the Cousa objective, 70 mW power was required for imaging the cells with the Nikon objective. The Cousa objective revealed IHCs responding to the 8 kHz sound stimulation (Fig. 4b). However, we were unable to observe any responsive cells with the Nikon objective (Fig. 4b). With the Nikon objective, signals gradually diminished during recording, likely due to the higher laser power used. To examine that possibility, bleaching was induced by the laser powers required for imaging with the Cousa or the Nikon objectives (Fig. 4b). The intensity of fluorescent particles decreased rapidly with the Nikon objective and 70 mW power, but were stable with the Cousa objective and 30 mW of power. Taken together, the Cousa objective enabled, to the best of our knowledge, the first in vivo two-photon calcium imaging of cochlear hair cells, owing to its unique combination of ultra-long WD and optimization for two-photon calcium imaging.

#### In vivo three-photon imaging across a large FOV

**Extended Data Fig. 10:**
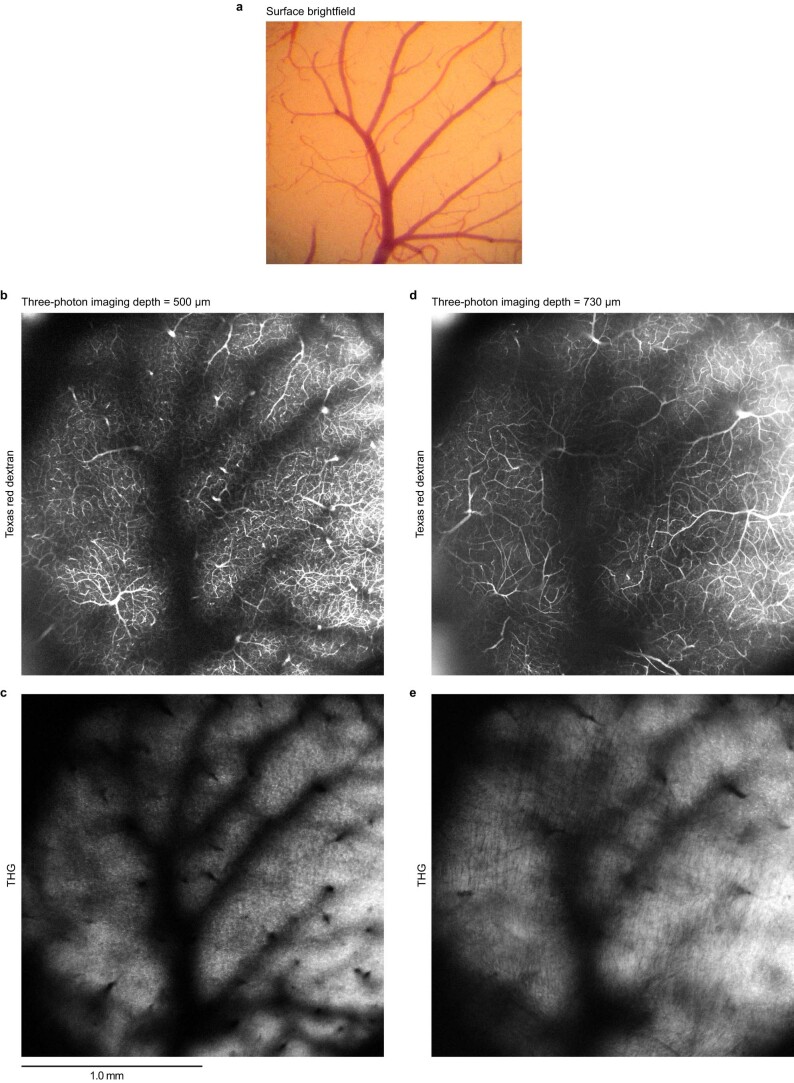
Large field-of-view three-photon imaging with the Cousa objective. **(a)** Brightfield image of a craniotomy over the mouse visual cortex, viewed through a surgical dissecting microscope. No dyes were present in the circulation at this phase of the experiment. Arteries and veins can be distinguished based on small difference in hue—arteries shown in orange and veins in purple. **(b,c)** Single z plane three-photon images (dual channel recording) from mouse visual cortex, 500 µm below the pial surface showing blood vessels labeled with Texas Red dextran (panel b) and THG signals (panel c). **(d, e)** Single z plane three-photon images (dual channel recording) from mouse visual cortex, 730 µm below the pial surface showing blood vessels labeled with Texas Red dextran (panel d) and THG signals (panel e).

Next, we used the Cousa objective for three-photon imaging. The Cousa supported three-photon imaging across one of the largest FOV^30^. We performed three-photon imaging of blood vessels, apical dendrites and white matter axons that were located between 500 and 1,000 µm from the pial surface in mouse visual cortex (Fig. 4c and Supplementary Video 3). Vessels were labeled with Texas Red dextran, apical dendrites and white matter axons were discernible with label-free THG (Methods)^31–38^. Even fine caliber blood vessels such as capillaries were clearly visible in cortical layers 5/6 and within the white matter (Fig. 4c). Moreover, in layer 5/6 apical dendrites were clearly represented as bright puncta in the THG channel (Fig. 4c, Extended Data Fig. 10 and Supplementary Video 3). In the white matter, parallel bands of axonal fibers were visible with THG (Fig. 4c, Extended Data Fig. 10 and Supplementary Video 3). The detection of fine-scale structural features located deep in the tissue (puncta of apical dendrites, orientated axonal fibers, capillaries) by the Cousa objective demonstrates performance reminiscent of conventional high NA, short WD water-dipping objectives, for example, the XLPLN25XWMP2 (Olympus; NA 1.05, WD 2 mm)^31–33^. Thus, the Cousa provides three-photon and THG imaging that compares well with conventional objectives, and the Cousa provides a seven-fold larger FOV area (roughly 2,000 × 2,000 µm^2^ versus 750 × 750 µm^2^).

#### In vivo two-photon calcium imaging in marmoset

**Fig. 5:**
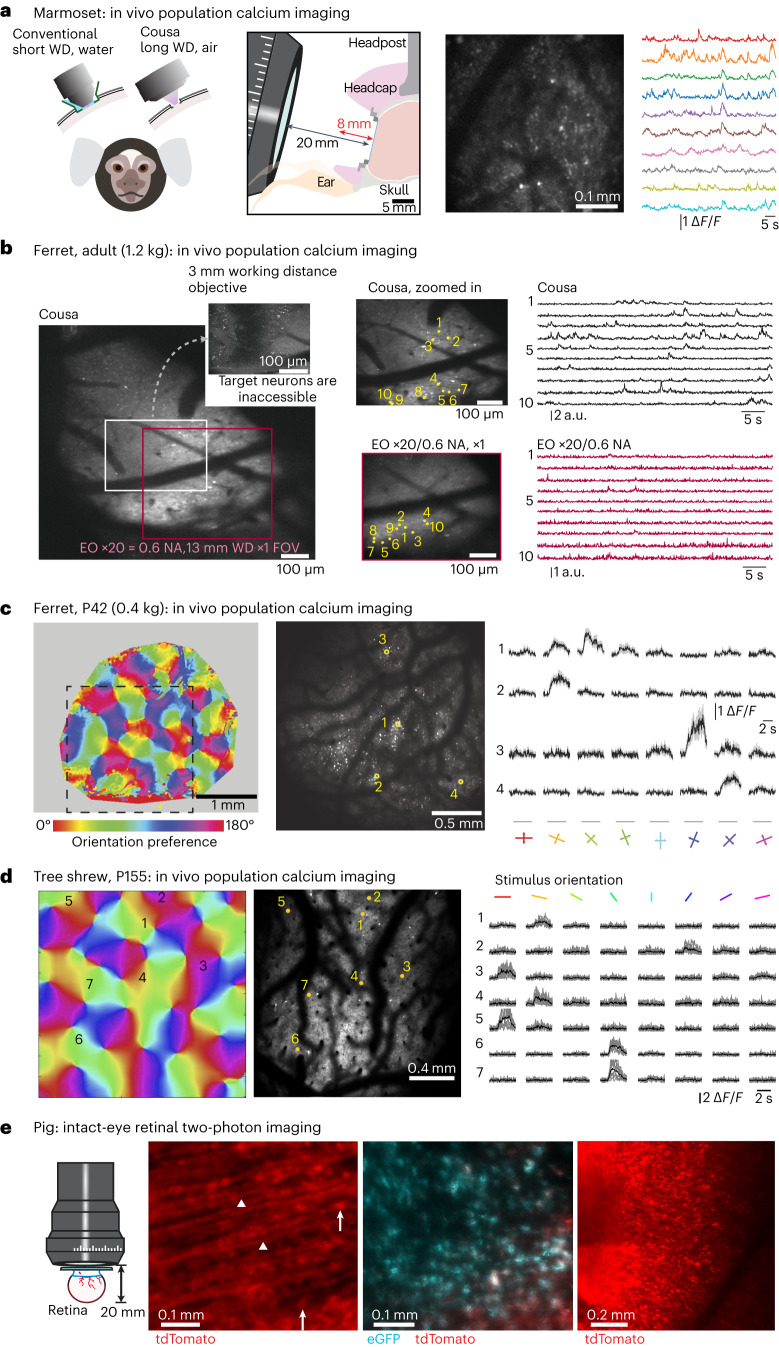
Two-photon imaging in larger mammals. **a**, In behaving marmosets, the Cousa objective facilitates imaging by replacing water immersion with air and providing ample open space around the cranial window. **b**, In ferrets, it can be challenging to access the neurons of interest. A short WD objective (Nikon ×16/0.8 NA, 3 mm WD in water) cannot even access the neurons of interest due to the short WD and collisions with the walls of the cranial window. The Cousa provides a large FOV and activity measurement, another long WD air objective provides only a smaller FOV (at a different angle) and weaker signals. Imaging depth is roughly 300 µm. **c**, In ferrets, with another type of imaging window, the Cousa provides a large FOV for measuring orientation tuning across orientation columns. **d**, Similarly, in tree shrew, the Cousa provides a large FOV for resolving individual neurons across orientation domains. **e**, In a challenging preparation, the Cousa enabled two-photon imaging through the lens and entire eye to the retina of an intact porcine eye, including retinal ganglion cell bodies (arrows) and single axon fibers (triangles).

The implants and optical windows for two-photon microscopy in awake monkeys are bulky and short WD objectives can collide with them and reduce the volume of tissue that is optically accessible. Thus, a long WD objective can be beneficial. Additionally, monkeys can perform tasks more reliably for longer periods of time when they are comfortably upright. In this position, the microscope objective often must approach the brain at an angle, and this complicates the maintenance of a liquid immersion interface. Thus, an air-immersion objective can facilitate or enable experiments (Fig. 5a). We tested the Cousa objective in a marmoset monkey. Two-photon imaging through the Cousa objective was used to resolve neuronal activity of individual neurons expressing jGCaMP7s (ref. ^13^) (Fig. 5a). In these marmoset imaging experiments, the setup time was shorter than for water immersion objectives, resulting in more time spent on imaging. The imaging was also more consistent even at extreme cranial window angles because the air immersion eliminated disruptions caused by unstable water or ultrasound gel interfaces or by air bubbles. These experiments show that the Cousa objective enhances multiphoton imaging experiments in awake marmosets.

#### In vivo two-photon calcium imaging in ferret and tree shrew

Imaging neuronal activity in ferrets and tree shrews can pose challenges for short WD water immersion objectives. Thus, we tested the Cousa objective for imaging in these animals. Two laboratories performed experiments in ferrets. In adult ferrets, the skull is thick and has prominent crests or ridges that result in cranial windows with significant geometric constraints. The use of a short WD objective is impaired by mechanical collisions with the walls surrounding the window. An objective with a 13 mm WD provided some optical access to the preparation. However, it was still not complete and the imaging angle had to be altered to accommodate the objective. Even then, the imaging quality was relatively poor, especially compared to the Cousa objective that provided better access, a larger FOV and better data quality (Fig. 5b).

For young ferret imaging, wide-field imaging was used to image the vasculature and the orientation preference map in V1 (Fig. 5c). Then, two-photon imaging through the Cousa objective was used to resolve neuronal activity of individual neurons within a 2 × 2 mm^2^ FOV (Fig. 5c). Individual neurons exhibited reliable responses to visual stimuli with edges of particular orientations (Fig. 5c). Observed two-photon orientation preferences were also consistent with their location within the orientation preference map measured with wide-field imaging. A similar approach was used for tree shrews (Fig. 5d). Again, individual neurons could be registered to their location in the local orientation preference map (Fig. 5d) and reliable responses to visual stimuli were resolved (Fig. 5d). In both ferret and tree shrew V1 imaging, the Cousa objective offered a larger FOV than conventional objectives and the air-immersion facilitated imaging, especially at angles where maintenance of a water interface can be unreliable. Together, these experiments demonstrate that the Cousa objective facilitates multiphoton imaging in tree shrews, and both young and adult ferrets.

#### Imaging the porcine eye

In studies of the neural circuitry of the porcine eye, high resolution imaging of fluorescently labeled cells typically requires the excision of the retina, which disrupts circuitry and precludes longitudinal studies. We used the Cousa objective to image cells and axons in the intact porcine eye, despite its roughly 20 mm diameter. After viral labeling, the intact eye was placed under the objective with hydroxypropyl methylcellulose gel (Ocular Vision) to match the corneal refractive index, and a coverslip (Fig. 5e). We imaged retinal ganglion cell cell bodies and axonal fibers, which have a diameter of roughly 1 µm (Fig. 5e). We identified an area of high enhanced green fluorescent protein (GFP) and tdTomato colocalization of retinal ganglion cell somas (Fig. 5e), demonstrating the ability of the objective and microscope system to image green and red fluorophores simultaneously. We then imaged an area of 1 × 1 mm^2^ to visualize blood vessel patterns for registering with postdissection images (Fig. 5e). The Cousa objective enabled direct imaging of the porcine retina through the intact eye, resolving individual cells and axon fibers.

## Discussion

In summary, we developed a microscope objective optimized to enable and enhance certain challenging multiphoton imaging experiments. The key attribute of the Cousa is its 20 mm WD in air. It is unique in that it combines this long WD with an NA of 0.50, optimization for multiphoton imaging and a long effective focal length, which provides a FOV of 4 mm^2^ (up to 9 mm^2^ at ±5° scanning). The manufactured objective has been distributed to an array of laboratories, and their results demonstrate functional and structural two-photon, three-photon and THG imaging in vivo. While short WD objectives remain an excellent choice for conventional experiments, there are several noteworthy results we presented here that were enabled or enhanced by the Cousa objective, including, to the best of our knowledge, the first in vivo two-photon calcium imaging in cochlear hair cells and the first two-photon imaging of porcine retina through the intact eye. We also presented one of the largest FOV for three-photon and THG imaging in vivo, resolving fine-scale structures such as apical dendrites and bundles of axons, with an ultra-long WD.

Previous development of an objective such as the Cousa was probably impaired by multiple issues. First, conventional microscopes have a mechanical envelope in which the microscope objective must fit, so that they do not collide with microscope stages and other components^17^. In this work, we recognized that the field of multiphoton imaging in vivo has evolved to support a range of imaging systems, many with no turrets or substage optics, that can accommodate objectives with mechanically larger form factors. Second, conventional microscopy has prioritized broad chromatic corrections and high NAs, and placed a lower priority on WD and balanced performance across a large FOV^17,39^. Thus, the area of the parameter space where the Cousa lies is relatively underexplored. Third, the potential applications for an ultra-long WD multiphoton objective were unclear. In this study, we show an array of applications that are enabled or enhanced by using a multiphoton-optimized ultra-long WD objective, including complex surgical preparations and applications with auxiliary optics in the post-objective space, including prisms and gradient index lenses. The Cousa can also enable experiments that entail multiphoton imaging simultaneous with electrophysiology using electrode arrays^40^ or patch clamp electrodes^41,42^ where short WD objectives would collide with the electrodes.

The lens description is open source, so that the community can replicate, modify or simulate for their applications. It could be promising to explore various engineering tradeoffs, including WD, mechanical size, NA and chromatic corrections. The objective we present here demonstrates the broad use and impact of alternative optical designs that depart from conventional parameters and constraints.

## Methods

### Objective design and assembly

The objective was modeled and optimized using an optical design software of OpticStudio (Zemax, LLC, v.22.2.1). Tolerance analysis indicated that 90% of the completed objectives would have an r.m.s. wavefront error of 0.048*λ* (still well below the diffraction limit criterion of 0.072*λ*) with commonly attained manufacturing and assembly tolerances. All lenses in the objective were manufactured, aligned and assembled in the factory of Special Optics. The manufacturing tolerances used were 0.005 mm total indicator runout for decentration and tilt, 0.05 mm for thickness, four rings for radius (power), 0.25 waves at 633 nm for irregularity, 0.005 mm for wedge, 60-40 scratch-dig and 0.01 arcmin for lens decentration.

### Custom in vivo two-photon imaging system

Two custom two-photon systems were used for most of the characterization and the mouse experiments in Figs. 1, 2 and 3a,b. One system was equipped with an 8 kHz resonant scanner (CRS 8 kHz, Cambridge Technology) and dual galvo scanners and supplied a 32-mm diameter beam size and ±5° scan angles at the objective back aperture^15^. The other system was equipped with a 12 kHz resonant scanner (CRS 12 kHz, Cambridge Technology) and supplied a higher imaging frame rate with a 20-mm beam size and ±2.6° scan angles at the objective back aperture. Our laser source was a Ti:sapphire pulsed laser with a central wavelength at 910 nm and an 80 MHz repetition rate (Mai-Tai). The image acquisition was controlled by ScanImage (Vidrio Technologies). The imaging was performed with a power less than or equal to 80 mW out of the front of the objective. Other imaging systems were used for the data in Figs. 4 and 5 and are detailed in the ‘Animal experiments’ section below.

### Excitation PSF measurements and simulations

The measurement and analysis procedure were described in our previous publication in detail^16^. To evaluate the excitation PSF, submicrometer beads were imaged. Submicrometer fluorescent beads (0.2 µm, Invitrogen F-8811) were embedded in a thick (roughly 1.2 mm) 0.75% agarose gel. Next, 30 µm *z* stacks were acquired, each centered at a depth 350 µm. The stage was moved axially in 0.5 µm increments (Δstage). At each focal plane 30 frames were acquired and averaged to yield a high signal-to-noise image.

The actual focal position within a specimen is different from what might be determined from objective or stage or sample movement due to the index mismatch between the air and the tissue. Due to the difference between the refractive index of the objective immersion medium (air) and the specimen medium (water), the actual focal position within the specimen was moved an amount Δfocus = 1.38 × Δstage (ref. ^44^). The factor 1.38 was determined in Zemax and slightly differs from the paraxial approximation of 1.33. These *z* stack images were imported into MATLAB for analysis. For the axial PSF, *xz* and *yz* images were created at the center of a bead, and a line plot was made at an angle maximizing the axial intensity spread, thereby preventing underestimation of the PSF due to tilted focal shifts. For the radial PSF, an *xy* image was found at the maximum intensity position axially. A line scan in *x* and *y* was made. Gaussian curves were fit to the individual line scans to extract FWHM measurements. The radial PSF values are an average of the *x* and *y* PSFs, and the axial PSF is an average of the axial PSF found from the *xz* and *yz* images. Excitation PSF measurements were performed at locations of on axis, 1°, 2° and 3° off axis across the FOV. Data reported (Fig. 1c) are the mean ± s.d. of five beads (*n* = 5) at each location.

The theoretical calculation of the PSF is based on the equations as follows, and can be converted into FWHM by multiplying $2\sqrt{\mathrm{ln}2}$ (ref. ^22^).

$${\omega }_{{xy}}=\left\{\begin{array}{c}\frac{0.320\lambda }{\sqrt{2}{\mathrm{NA}}}\,{\rm{and }}\,{\mathrm{NA}}\le 0.7\\ \frac{0.325\lambda }{{\sqrt{2}{\mathrm{NA}}}^{0.91}}\,{\rm{and }}\,{\mathrm{NA}} > 0.7\end{array}\right.$$

$${\omega }_{z}=\frac{0.532\lambda }{\sqrt{2}}\left[\frac{1}{n-\sqrt{{n}^{2}-{\mathrm{NA}}^{2}}}\right]$$

where *n* is the refractive index of the medium where the sample is embedded and *λ* is the excitation wavelength. Using the value of 910 nm for the excitation wavelength, 0.5 for the NA and 1.33 for the refractive index of water, we have FWHM_*xy*_ = 0.69 µm and FWHM_*z*_ = 5.84 µm.

The broadband antireflective coating applied to the lenses was measured to transmit on average 99.5% of visible and near-infrared light (450–1,100 nm) per surface. To measure the total transmission of 910 and 532 nm light through this objective, we supplied an under-filling laser beam into the objective and measured its power before and after the objective.

### Animal experiments

#### Mouse population calcium imaging and dendritic calcium imaging

All procedures involving living animals for these figures were carried out in accordance with the guidelines and regulations of the US Department of Health and Human Services and approved by the Institutional Animal Care and Use Committee at University of California, Santa Barbara. Mice were housed in 12 h dark/light reverse cycle room. The temperature set-point is 23–24 °C (74–76 °F); the low-temperature alarm is 21 °C (70 °F); the high-temperature alarm is 25.5 °C (78 °F). The relative humidity is 45% (range 30–70%).

For population calcium imaging, GCaMP6s transgenic mice were used as before^45^, which were generated by triple crossing of TITL-GCaMP6s mice, Emx1-Cre mice (Jackson Laboratories stock no. 005628) and ROSA:LNL:tTA mice (Jackson Laboratories stock no. 011008). TITL-GCaMP6s mice were kindly provided by the Allen institute. Transgenic mice were deeply anesthetized using isoflurane (1.5–2%) augmented with acepromazine (2 mg kg^−1^ body weight) during craniotomy surgery. Carpofen (5 mg kg^−1^ body weight) was administered before as well as after surgery for three consecutive days. Glass windows were implanted over visual cortex as previously described^15,45^. Neurons were segmented and fluorescence time courses of Ca^2+^ signals were extracted from imaging stacks using Suite2p (https://suite2p.readthedocs.io/en/latest/)^46,47^. Signals from neurons are a sum of neuronal and neuropil components. The neuropil component was subtracted from the neuronal signals by separately detecting it and subtracting it. The neuropil component was isolated using the signal from an annulus region around each neuron, and then subtracted from the neuronal signal to provide a higher fidelity report of neuronal fluorescence dynamics. An exponential moving average with a moving window size of five samples (0.32 s) was used to reduce the baseline noise in the traces displayed (Fig. 3a).

For dendrite calcium imaging, adult (more than 8 weeks) C57Bl/6 mice of both sexes (Jackson Laboratories) were used. A 4-mm diameter craniotomy was performed over visual cortex as previously described^48^. Briefly, mice were premedicated with a sedative, acepromazine (2 mg kg^−1^ body weight, i.p.), after which they were deeply anesthetized using isoflurane (2–3% for induction, 1–1.5% for surgery). The mouse’s body temperature was monitored and actively maintained using an electronic heat pad regulated via rectal probe. Carprofen (5 mg kg^−1^ body weight subcutaneous) was administered preoperatively, and lidocaine solution containing epinephrine (5 mg kg^−1^ body weight subcutaneous) was injected locally before and after the scalp excision. The scalp overlaying the right visual cortex was removed and a custom head-fixing imaging chamber with a 5-mm diameter opening was mounted to the skull with cyanoacrylate-based glue (Oasis Medical) and dental acrylic (Lang Dental). Mice were mounted on a custom holder via the headplate chamber, which was filled with a physiological saline containing (in mM) 150 NaCl, 2.5 KCl, 10 HEPES, 2 CaCl_2_ and 1 MgCl_2_. A craniotomy was performed using carbide and diamond dental burs on a contra-angle handpiece (NSK). adeno-associated viral (AAV) vectors were injected in to V1 under continued isoflurane anesthesia as previously described^48–50^. Briefly, 1:1 mixture of pENN.AAV.CamKII 0.4.Cre.SV40(AAV1; Addgene catalog no. 105558; diluted at 1:20,000 in phosphate buffered saline) and pGP.AAV.syn.FLEX.jGCaMP8m.WPRE (AAV1; Addgene catalog no. 162378; original concentration at roughly 10^13^ vg ml^−1^) viral particles were injected (80 nl per site; ONE site per animal) into V1 with a pulled-glass capillary micropipette using a Nanoliter 2010 controlled by a microprocessor, Micro4 (World Precision Instruments), at 15 nl per min. The glass pipette was left in place for 5 min before retracting to avoid the backflushing of the injected solution. The cranial window was then sealed with a glass cranial plug made up of 4 and 3 mm circular coverslips (Warner Instruments) stacked in tandem with a UV-curing optical adhesive (catalog no. NOA61, Norland). Two-photon imaging of Ca^2+^ transients indicated by GCaMP8m was performed starting 4–6 weeks after AAV injection, using a custom-built two-photon microscope used in previous studies^41,48^. Frame scans were acquired using ScanImage^51^ at 58.2 frames per second, 512 × 256 pixels; 31,000 frames total per visual stimulation session.

Visual stimuli were presented on a 7 inch monitor (60 Hz refresh rate) placed 12 cm away from the animal’s eye. To assess orientation tuning of the dendritic shaft, spines and putative axonal boutons, full field square gratings at 40, 70 and 100% contrasts (0.04 cycles per degree at 2 Hz) were presented in eight directions (0°, 45°, 90°, 135°, 180°, 225°, 270°, 315°) for five trials. Each grating drifted for 4 s. A notch filter centered at 2 Hz (±0.5 Hz bandwidth) was used to remove a small amount of light leakage from the stimulus monitor into the imaging pathway.

To functionally map visual cortex for targeted injection of viral vectors, ISOI was performed using a custom macroscope and a CCD camera as previously described^48,52^. Retinotopic maps were used to locate V1. The pial vasculature map relative to the retinotopic maps was used to guide targeted injections into V1.

#### Mouse cochlea imaging

Animal studies were carried out according to the protocols approved by the Institutional Animal Care and Use Committee at Stanford University (APLAC-14345). Four-week-old male mouse from Ai14tdTomato (JAX: 007908) × Myosin15Cre (ref. ^53^) breeding was used for cochlear hair cell imaging. The mouse was anesthetized using ketamine (100 mg kg^−1^) and xylazine (10 mg kg^−1^). Anesthesia level was assessed by signs of movement or withdrawal reflex before the application of supplementary anesthetic. Mouse surgery and positioning for in vivo cochlear imaging was performed by the method described previously^29^. In vivo cochlear imaging was performed using a modified commercial two-photon microscope (Ultima, Bruker) with long WD air objectives (Cousa objective; TU Plan ELWD ×20, NA 0.4, WD 19 mm, Nikon Instruments Inc.). A Ti:sapphire laser was used with wavelength 920 nm and power 30 mW (Chameleon, Coherent Inc.) at the exit of the objective. The projected images (Fig. 4b) were acquired in an apical hair cell location (8–10 kHz) by collecting *z* series 40 images with 2 µm intervals.

#### Mouse prism-based two-photon imaging with simultaneous wide-field imaging

All animal handling and experiments were performed according to the ethical guidelines of the Institutional Animal Care and Use Committee of the Yale University School of Medicine. Brain-wide expression of GCaMP6s was achieved via neonatal sinus injection of AAV9-Syn-GCaMP6s into c57/Bl6 mice, as described previously^10,54^. After reaching adulthood (P60), the skin and fascia over the skull were removed under isoflurane anesthesia and the animal was implanted with a custom titanium headpost and a microprism (5 mm per side, Tower Optics) placed over the right visual cortex in a small craniotomy, bonded with a thin layer of dental cement (Metabond, Parkell).

Imaging experiments were carried out in awake mice head-fixed over a freely moving wheel placed under the microscope objective. Wide-field calcium imaging was performed using a Zeiss Axiozoom with a PlanNeoFluar objective (×1, 0.25 NA). Epifluorescent excitation was provided by an LED bank (Spectra X Light Engine, Lumencor) strobing 395 and 470 nm light, for hemodynamic correction and calcium imaging, respectively^54^. Emitted light was collected via sCMOS camera (Orca-Flash V3, Hamamatsu), with images acquired at 512 × 512 pixel resolution and 10 frames per second. Data were preprocessed for hemodynamic correction and normalized to Δ*F*/*F* values as previously described^2^. Functional parcellation of cortical areas was carried out using local selective spectral clustering^49,50^ to obtain a time series of fluorescence signal for each parcel^55^.

Two-photon imaging was performed using a resonant-galvo scanning microscope (MOM, Sutter Instruments) coupled to our custom air-coupled, long WD objective (×10, 0.5 NA). Excitation was provided by a titanium-sapphire laser (Mai-Tai, Spectra-Physics) tuned to 920 nm. Light was directed into the brain after being reflected 90^◦^ by the implanted prism. Emitted light was collected by a gallium arsenide-phosphide detector (Hamamatsu) with images acquired at 512 × 512 pixel resolution and 30 frames per second. Data were motion corrected using NoRMCorre^56^, and regions of interest (ROI) corresponding to single cells were manually selected, neuropil-corrected and normalized to Δ*F*/*F* values using procedures written in MATLAB (MathWorks).

We calculated cell-centered networks to quantify the relationship between activity in single neurons and the large-scale cortical network in the contralateral hemisphere as described previously^10^. Briefly, we evaluated the correlation coefficients between time series related to *p* mesoscopic parcels and *n* time series related to cells to obtain *C*, a *p* × *n* matrix. We viewed each column of *C* as a compact representation of synchrony between the dynamics of each cell and the dynamics of the wide-field signal and then clustered these vectors using the kmeans (*k* = 4) function in MATLAB. We obtained the centroid map of each cluster as the average correlation coefficients of all cells related to a specific cluster.

We then superimposed each centroid onto the full cortex parcellation to yield the average images in Fig. 4a.

#### Mouse three-photon imaging

All animal procedures were approved by the Institutional Animal Care and Use Committee at the University of Minnesota. Our surgical and imaging methods have been described previously^32,57,58^. Briefly, in testing the Cousa objective lens for deep tissue three-photon imaging, two C57BL/6J mice were used. Mice were initially anesthetized with a bolus injection of fentanyl citrate (0.05 mg kg^−1^), midazolam (5 mg kg^−1^), and dexmedetomidine (0.25 mg kg^−1^). A craniectomy (3–4 mm in diameter) was made over the visual cortex. The cranial windows were sealed with agarose (1.5% in artificial cerebrospinal fluid) and a glass coverslip (5 mm diameter, 0.15 mm thickness; Warner catalog no. D263). During imaging, continuous intraperitoneal infusion with a lower concentration mixture (fentanyl citrate 0.002–0.03 mg kg^−1^ h^−1^, midazolam 0.2–3.0 mg kg^−1^ h^−1^ and dexmedetomidine 0.010–0.15 mg kg^−1^ h^−1^) was administered using a catheter connected to a syringe pump. Three-photon imaging was performed using a Bruker Ultima Investigator Microscope coupled to a fixed wavelength laser source from Class 5 Photonics. The excitation wavelength was fixed at 1,300 nm using a White Dwarf WD-1300-80-60 femtosecond optical parametric chirped pulse amplifier pumped by a 0.5 MHz Coherent Monaco-1035-80-60 laser. The Cousa objective lens correction collar was set to 0.15 mm to compensate for the aberration from the coverslip. Three-photon brightness was maximized using a motorized single-prism pulse compressor (BOA-1300, Swamp Optics). To visualize blood vessels, we injected Texas Red dextran retro-orbitally^32^. In addition, we used label-free THG (excitation at 1,300 nm) to visualize apical dendrites and white matter axons^31,32^. This is because THG is an intrinsic signal^34^ that is generated from three-photon excitation and it provides label-free contrast at junctions of biological materials with dissimilar nonlinear indices of refraction, for example, at lipid boundaries^35^ or elastic fibers^38^. More generally, THG signals have found a variety of applications from mapping the structure of white matter axonal tracts and neuronal cell membranes^31,32^, to tracking blood flow dynamics^36,37^.

#### Marmoset experiments

All experimental procedures were approved by The Rockefeller University Institutional Animal Care and Use Committee and were performed in accordance with guidelines from the US National Institute of Health. One 4-year-old marmoset participated in this study.

A headpost and headcap were first implanted, followed by implantation of a 10 mm diameter cranial window over temporal cortex. The cover glass was 0.17 mm in thickness. Viral transduction was accomplished with microinjection of a solution containing a 1:1 ratio of AAV2/9:Thy1S-tTA (Vigene Biosciences, 6.57 × 10^13^ gc ml^−1^) and AAV2/9:TRE3G- jGCaMP7s-WPRE (Vigene Biosciences, 6.20 × 10^13^ gc ml^−1^)^59^. The solution had a titer of 1 × 10^13^ vg ml^−1^ for each virus and was injected through glass micropipettes (tip diameter 40–60 µm, 30° bevel) in a volume of 500 nl at 0.5 mm and again at 1.0 mm depths. Thirteen locations across the window were injected at a rate of 100 nl min^−1^ with a 10 min pause before retracting the micropipette from each site.

The marmoset was placed into a custom-made chair and head fixation established using a custom-designed triangular headpost fit into a custom-machined clamp. Two-photon imaging of jGCaMP7s was performed with a custom-built microscope equipped with a 12 kHz resonant-galvo scanner and driven by a Spectra-Physics Mai-Tai DeepSee laser tuned to 920 nm at a frame rate of 45 Hz or with a Thorlabs Multiphoton Mesoscope driven by a Class 5 Photonics White Dwarf laser tuned to 960 nm at a frame rate of 6.36 Hz. Average excitation power at the exit of the objective ranged from 50 to 80 mW. The objective was angled 70–90° relative to the body axis of the marmoset. Microscopes were controlled by ScanImage (MBF Bioscience). Images were acquired in the absence of controlled stimuli. Neuron ROI locations and fluorescence time courses were extracted from the resulting image stacks using Suite2p (https://suite2p.readthedocs.io/en/latest/)^24^. Fluorescence traces for each ROI were calculated as the Δ*F*/*F* = (*F* − *F*_0_)/*F*_0_, where the baseline fluorescence (*F*_0_) was approximated as the mean fluorescence across the entire image stack.

#### Ferret and tree shrew experiments

All experimental procedures were approved by the Max Planck Florida Institute for Neuroscience Institutional Animal Care and Use committee and were performed in accordance with guidelines from the US National Institute of Health. We used one juvenile female ferret from Marshal Farms and one adult male tree shrew for this study.

Viral transduction and terminal imaging in L2/3 of the anesthetized ferret and tree shrew were performed as previously described^60,61^. Briefly, we expressed GCaMP6s by direct microinjection of AAV2/1-hSyn-GCaMP6s-WPRE-SV40 (Addgene, 100843-AAV1, titer 2.5 × 10^13^ GC ml^−1^) into the visual cortex. Subsequently a cranial window was implanted over visual cortex and imaged. An injection into the visual cortex of the ferret was made at P21, and imaging was performed at P42 (386 g body weight). Imaging in the tree shrew occurred 16 days after viral transduction.

Two-photon imaging of GCaMP6s was performed with a Bergamo II series microscope (Thorlabs) equipped with an 8 kHz resonant-galvo scanner and driven by a Mai-Tai DeepSee laser or Insight DS+ (Spectra-Physicis) at 910 or 920 nm. respectively. Average excitation power at the exit of the objective ranged from 40 to 60 mW. The microscope was controlled by ScanImage (MBF Bioscience). Images were acquired at 15 Hz (1,024 × 1,024 pixels in the ferret, 512 × 512 pixels in the tree shrew). Wide-field epifluorescence imaging of GCaMP6s in the ferret was achieved with a Zyla 5.5 sCMOS camera (Andor) controlled by µManager^62^ through a ×4 air-immersion objective (Olympus, UPlanFL ×4/0.13 NA) and images were acquired at 15 Hz with 4 × 4 binning to yield 640 × 540 pixel images.

Visual stimuli were presented on an LCD screen using PsychoPy (v.1.85)^63^. The monitor (30 × 52 cm^2^, 1,920 × 1,080 pixels, 120 Hz refresh rate) was placed 25 cm in front of the animal. To evoke orientation-specific responses, full field square gratings at 100% contrast were presented in 16 directions (eight orientations) for ten trials (ferret) or eight trials (tree shrew). Square gratings were presented to the ferret at 0.06 cycles per degree and 4 Hz and in the tree shrew at 0.4 cycles per degree and 2 Hz. In addition, ‘blank’ stimuli of 0% contrast were also presented. All stimuli were randomly interleaved and presented for 4 s followed by 6 s of gray screen (ferret) or 2 s followed by 3 s of gray screen (tree shrew). Timing for visual stimuli and imaging were recorded using Spike2 (v.7.11b, CED).

Data analysis in the ferret was performed as previously described using scripts in Python and ImageJ^64^. For both wide-field and epifluorescence imaging, we corrected brain movement during imaging by maximizing phase correlation to a common reference frame. In wide-field epifluorescence imaging, the ROI was drawn manually around regions where robust visually evoked activity was observed. For analysis, all images were spatially downsampled by a factor of two to yield 320 × 270 pixels. Slow drifts in fluorescence intensity were eliminated by calculating the Δ*F*/*F* = (*F* − *F*_0_)/*F*_0_. Baseline fluorescence (*F*_0_) was calculated by applying a rank-order filter to the raw fluorescence trace (tenth percentile) with a rolling time window of 60 s. Responses were filtered with a spatial band-pass filter with low-pass cutoff defined as 50 µm and high-pass filter cutoff as 3,200 µm. Preferred orientation was computed by taking the vector sum of the median-trial response over the stimulus period for each orientation.

For analysis, ROI were chosen semi-automatically (Cell Magic Wand v.1.0) and fluorescence was computed by averaging all pixels within the ROI^49^. The Δ*F*/*F* for each ROI was computed, and F0 was calculated by applying a rank-order filter to the raw fluorescence (20th percentile) over a rolling time window (60 s). Stimulus-evoked responses were calculated as the average Δ*F*/*F* over the entire stimulus period, and orientation preferences were computed by fitting a von Mises distribution to the trial-median response for each stimulus orientation.

Data analysis and motion correction in the tree shrew were performed using procedures written in MATLAB (MathWorks) or Java package for running ImageJ within MATLAB (Mĳi). For network-level analysis, the fluorescence signal for each pixel was calculated as Δ*F*/*F*, where *F*_0_ is the baseline fluorescence signal averaged over a 1 s period immediately before the start of visual stimulus and *F* is the fluorescence signal averaged over the period of the stimulus. Responses to the stimulus set were fitted with a Gaussian to determine the preferred orientation and generate a pixel-based orientation preference map. For analysis at the neuronal level, ROIs corresponding to visually identified neurons were drawn manually using ImageJ. The fluorescence of each ROI was measured by averaging all pixels within the ROI.

#### Adult ferret experiments with objective comparisons

All procedures adhered to the guidelines of the National Institute of Health and were approved by the Animal Care and Use Committee at Johns Hopkins University. The experiment was performed in an adult male ferret (*Mustela putoris* furo; 120 days old, 1.2 kg body weight). Virus was injected during an aseptic procedure under isoflurane anesthesia. During this procedure, a craniotomy of approximately 2 × 2 mm^2^ was made over V1. Virus (AAV1.syn.jGCaMP7s.WPRE.SV40 from Addgene, lot no. v50167, titer 2.7 × 10^13^ GC ml^−1^) was then injected at two sites within the durotomy. At each site, injections were performed at multiple depths for a total of about 1 μl per site. Then, 14 days after the procedure to allow for virus expression, we performed an anesthetized two-photon experiment using the same procedures as described previously^65^. Briefly, during the experiment, ferrets were induced with 40 mg kg^−1^ ketamine and 0.05 mg kg^−1^ atropine intramuscularly and maintained on isoflurane anesthesia, and paralyzed using pancuronium bromide (0.15 mg kg^−1^ h^−1^). Continuous monitoring of a range of vital parameters (heart rate, SpO_2_, electrocardiography, EtCO_2_ and electroencephalography) ensured adequate anesthetic depth during the experiment. A custom-made stainless steel imaging chamber was cemented to the skull centered on the virus injection site. The bone and dura over the virus injection site were then removed, the brain covered with a thin layer of agarose (type III, Sigma-Aldrich) and a coverslip. Imaging was performed at 920 nm using a Coherent Ti:Sapphire laser coupled to a two-photon microscope from Neurolabware.

#### Porcine eye experiments

The study was conducted with approval by the Administrative Panel for Laboratory Animal Care at Stanford University and in accordance with the Guide for the Care and Use of Laboratory Animals at an AAALAC-accredited facility. A 6-month-old female Yucatan minipig was obtained from an approved vendor (Premier BioSource), acclimated for at least 3 days and group housed under standard conditions.

Before anesthesia, the animal was fasted for 12–18 h. The animal was sedated with ketamine (5 mg kg^−1^), dexmedetomidine (0.03 mg kg^−1^) and butorphanol (0.2 mg kg^−1^) intramuscularly and maintained with mask isoflurane (1–4%) in 100% oxygen. Heart rate, respiratory rate, blood oxygen saturation, body temperature and jaw tone were monitored continuously and recorded every 15 min.

The right eye was topically treated with tetracaine hydrochloride ophthalmic solution (0.5%). The right orbital area was clipped and aseptically prepared with saline and ophthalmic betadine (5%). Using sterile technique, a 30-gauge insulin syringe was used to aspirate 150 µl of aqueous fluid. With the guidance of Castroviejo calipers, AAV2-CAG-GFP (50 µl, 1.5 × 1,013 GC ml^−1^, Addgene catalog no. 37825-AAV2) was injected intravitreally 4 mm posterior to the temporal aspect of the limbus, and AAV2-CAG-TdTomato (100 µl, 5.3 × 1,012 GC ml^−1^, Addgene catalog no. 59462-AAV2) was injected intravitreally 4 mm posterior to the nasal aspect of the limbus. Neomycin, polymyxin B sulfate and bacitracin zinc ophthalmic ointment was topically applied to the eye. Anesthesia was reversed with atipamezole (0.35 mg kg^−1^) intramuscularly, and the animal recovered.

Seven weeks later, the animal was sedated with Telazol (6 mg kg^−1^) intramuscularly and euthanized with pentobarbital sodium and phenytoin (8 ml; 390 and 50 mg ml^−1^) IV. Subsequently, the right eye was harvested for two-photon imaging.

Two-photon imaging was performed using the 20 mm WD objective on an Ultima IV two-photon microscope (Bruker) equipped with two GaAsP PMTs and a custom fiber nosepiece. Laser excitation consisted of a variable wavelength laser set to 920 nm, and a steady state laser at 1,040 nm (Spectra-Physics). The enucleated eye was placed under the objective with hydroxypropyl methylcellulose gel (Ocular Vision) and a 1.5 coverslip on top.

### Statistics and reproducibility

The number of independent repeats (*n*) with similar results for each in vivo experiments are as follows: *n* = 2 for Fig. 2e; *n* = 2 for Fig. 3a; *n* = 2 for Fig. 3b; *n* = 1 for Fig. 4a; *n* = 2 for Fig. 4b; *n* = 2 for Fig. 4c; *n* = 2 for Fig. 5a; *n* = 2 for Fig. 5b; *n* = 1 for Fig. 5c; *n* = 1 for Fig. 5d; *n* = 1 for Fig. 5e and *n* = 2 for Extended Data Figs. 8 and 10.

### Reporting summary

Further information on research design is available in the Nature Portfolio Reporting Summary linked to this article.

## Online content

Any methods, additional references, Nature Portfolio reporting summaries, source data, extended data, supplementary information, acknowledgements, peer review information; details of author contributions and competing interests; and statements of data and code availability are available at 10.1038/s41592-023-02098-1.

### Supplementary information

Reporting Summary

Supplementary Video 1 *Z* stack of in vivo calcium imaging. The *z* plane range spans from the brain surface to the depth of 500 µm. Frame size is 1,024 × 1,024 pixels. Imaging power at the imaging plane is 80 mW.

Supplementary Video 2 In vivo calcium imaging over a 1.7 × 1.7 mm^2^ FOV. Frame rate is 15.4 frames per s. Frame size is 1,536 × 1,536 pixels. Imaging power at the imaging plane is 60 mW. Imaging depth is 250 µm.

Supplementary Video 3 In vivo three-photon imaging *z* stack from the mouse visual cortex and white matter below using the Cousa objective lens. The *xy* FOV shown represents a region of 0.7 × 0.7 mm^2^. The depth range spans 0.270 to 0.945 mm from the pial surface and thus starts in cortical layer 2/3 and ends below the white matter. The lumen of blood vessels is visible (shown in magenta) after a retro-orbital injection of Texas Red dextran. Apical dendrites (distinct puncta) and white matter axonal fibers are visible with THG (shown as cyan).

## Data Availability

The design of the objective is fully open source and full specification are detailed in this report. The datasets reported here are openly available in figshare at 10.6084/m9.figshare.24164142.

## Code availability

The code used in this work is already publicly available or detailed in Methods. If additional materials are required for replication, the authors invite such requests.

## References

[1] Trautmann EM (2021). Dendritic calcium signals in rhesus macaque motor cortex drive an optical brain-computer interface. Nat. Commun..

[2] Heider B, Nathanson JL, Isacoff EY, Callaway EM, Siegel RM (2010). Two-photon imaging of calcium in virally transfected striate cortical neurons of behaving monkey. PLoS ONE.

[3] Li M, Liu F, Jiang HF, Lee TS, Tang SM (2017). Long-term two-photon imaging in awake macaque monkey. Neuron.

[4] Macknik SL (2019). Advanced circuit and cellular imaging methods in nonhuman primates. J. Neurosci..

[5] Smith GB, Fitzpatrick D (2016). Viral injection and cranial window implantation for in vivo two-photon imaging. Methods Mol Biol..

[6] Trautmann, E. et al. Design of an implantable artificial dural window for chronic two-photon optical imaging in non-human primates. In *Proc. 37th Annual International Conference of the IEEE Engineering in Medicine and Biology Society (EMBC)* 7554–7557 (IEEE, 2015). 10.1109/EMBC.2015.7320140 PMC8867469 26738040

[7] O’Shea DJ (2017). The need for calcium imaging in nonhuman primates: new motor neuroscience and brain-machine interfaces. Exp. Neurol..

[8] Huang L, Merson TD, Bourne JA (2016). In vivo whole brain, cellular and molecular imaging in nonhuman primate models of neuropathology. Neurosci. Biobehav. Rev..

[9] Terada S-I, Kobayashi K, Ohkura M, Nakai J, Matsuzaki M (2018). Super-wide-field two-photon imaging with a micro-optical device moving in post-objective space. Nat. Commun..

[10] Barson D (2020). Simultaneous mesoscopic and two-photon imaging of neuronal activity in cortical circuits. Nat. Methods.

[11] Redman WT (2022). Long-term transverse imaging of the hippocampus with glass microperiscopes. eLife.

[12] Chen T-W (2013). Ultrasensitive fluorescent proteins for imaging neuronal activity. Nature.

[13] Dana H (2019). High-performance calcium sensors for imaging activity in neuronal populations and microcompartments. Nat. Methods.

[14] Zhang, Y. et al. jGCaMP8 fast genetically encoded calcium indicators. *Janelia Research*10.25378/janelia.13148243.v4 (2020).

[15] Yu C-H, Stirman JN, Yu Y, Hira R, Smith SL (2021). Diesel2p mesoscope with dual independent scan engines for flexible capture of dynamics in distributed neural circuitry. Nat. Commun..

[16] Stirman, J. N., Smith, I. T., Kudenov, M. W. & Smith, S. L. Wide field-of-view, multi-region, two-photon imaging of neuronal activity in the mammalian brain. *Nat. Biotechnol.* **34**, 857–85 (2016). 10.1038/nbt.3594 PMC4980167 27347754

[17] Zhang Y, Gross H (2019). Systematic design of microscope objectives. Part I: system review and analysis. Adv. Opt. Technol..

[18] Ji N, Freeman J, Smith SL (2016). Technologies for imaging neural activity in large volumes. Nat. Neurosci..

[19] O’Shea DC (2006). Group velocity dispersion using commercial optical design programs. Appl. Opt..

[20] Bobroff N, Rosenbluth AE (1992). Evaluation of highly corrected optics by measurement of the strehl ratio. Appl. Opt..

[21] Helmchen F, Denk W (2005). Deep tissue two-photon microscopy. Nat. Methods.

[22] Zipfel WR, Williams RM, Webb WW (2003). Nonlinear magic: multiphoton microscopy in the biosciences. Nat. Biotechnol..

[23] Tung, C.-K. et al. Effects of objective numerical apertures on achievable imaging depths in multiphoton microscopy. *Microsc. Res. Tech.* **65**, 308–314 (2004). 10.1002/jemt.20116 15662621

[24] Dunn AK, Wallace VP, Coleno M, Berns MW, Tromberg BJ (2000). Influence of optical properties on two-photon fluorescence imaging in turbid samples. Appl. Opt..

[25] Chen TW (2013). Ultrasensitive fluorescent proteins for imaging neuronal activity. Nature.

[26] Andermann ML (2013). Chronic cellular imaging of entire cortical columns in awake mice using microprisms. Neuron.

[27] Resendez SL (2016). Visualization of cortical, subcortical and deep brain neural circuit dynamics during naturalistic mammalian behavior with head-mounted microscopes and chronically implanted lenses. Nat. Protoc..

[28] Kim, J. & Ricci, A. J. A chemo-mechanical cochleostomy preserves hearing for the in vivo functional imaging of cochlear cells. *Nat. Protoc.* **18**, 1137–1154 (2023). 10.1038/s41596-022-00786-4 36599963

[29] Kim J, Ricci AJ (2022). In vivo real-time imaging reveals megalin as the aminoglycoside gentamicin transporter into cochlea whose inhibition is otoprotective. Proc. Natl Acad. Sci. USA.

[30] Mok, A. T. et al. A large field of view two- and three-photon microscope for high-resolution deep tissue imaging. In *CLEO 2023, Technical Digest Series* White Paper No. ATh5A.1 (Optica Publishing Group, 2023).

[31] Ouzounov DG (2017). In vivo three-photon imaging of activity of gcamp6-labeled neurons deep in intact mouse brain. Nat. Methods.

[32] Liu CJ, Roy A, Simons AA, Farinella DM, Kara P (2020). Three-photon imaging of synthetic dyes in deep layers of the neocortex. Sci. Rep..

[33] Hontani Y, Xia F, Xu C (2021). Multicolor three-photon fluorescence imaging with single-wavelength excitation deep in mouse brain. Sci. Adv..

[34] Squier JA, Müller M, Brakenhoff G, Wilson KR (1998). Third harmonic generation microscopy. Opt. Express.

[35] Débarre D (2006). Imaging lipid bodies in cells and tissues using third-harmonic generation microscopy. Nat. Methods.

[36] Witte S (2011). Label-free live brain imaging and targeted patching with third-harmonic generation microscopy. Proc. Natl Acad. Sci. USA.

[37] Cheng H (2022). Label-free measurement of wall shear stress in the brain venule and arteriole using dual-wavelength third-harmonic-generation line-scanning imaging. Opt. Lett..

[38] Yu C-H (2007). In vivo and ex vivo imaging of intra-tissue elastic fibers using third-harmonic-generation microscopy. Opt. Express.

[39] Tsai PS (2015). Ultra-large field-of-view two-photon microscopy. Opt. Express.

[40] Steinmetz NA (2021). Neuropixels 2.0: a miniaturized high-density probe for stable, long-term brain recordings. Science.

[41] Smith SL, Smith IT, Branco T, Häusser M (2013). Dendritic spikes enhance stimulus selectivity in cortical neurons in vivo. Nature.

[42] Kitamura K, Judkewitz B, Kano M, Denk W, Häusser M (2008). Targeted patch-clamp recordings and single-cell electroporation of unlabeled neurons in vivo. Nat. Methods.

[43] Voigt, F. F. et al. Reflective multi-immersion microscope objectives inspired by the Schmidt telescope. *Nat. Biotechnol.*10.1038/s41587-023-01717-8 (2023). 10.1038/s41587-023-01717-8 PMC10791577 36997681

[44] Visser TD, Oud JL (1994). Volume measurements in three-dimensional microscopy. Scanning.

[45] Yu Y, Stirman JN, Dorsett CR, Smith SL (2022). Selective representations of texture and motion in mouse higher visual areas. Curr. Biol..

[46] Yu, Y., Stirman, J. N., Dorsett, C. R. & Smith, S. L. Mesoscale correlation structure with single cell resolution during visual coding. Preprint at bioRxiv 10.1101/469114 (2019).

[47] Pachitariu, M. et al. Suite2p: beyond 10,000 neurons with standard two-photon microscopy. Preprint at bioRxiv 10.1101/061507 (2017).

[48] Smith IT, Townsend LB, Huh R, Zhu H, Smith SL (2017). Stream-dependent development of higher visual cortical areas. Nat. Neurosci..

[49] Wilson DE, Whitney DE, Scholl B, Fitzpatrick D (2016). Orientation selectivity and the functional clustering of synaptic inputs in primary visual cortex. Nat. Neurosci..

[50] Iacaruso MF, Gasler IT, Hofer SB (2017). Synaptic organization of visual space in primary visual cortex. Nature.

[51] Pologruto TA, Sabatini BL, Svoboda K (2003). ScanImage: flexible software for operating laser scanning microscopes. Biomed. Eng. Online.

[52] Kalatsky VA, Stryker MP (2003). New paradigm for optical imaging: temporally encoded maps of intrinsic signal. Neuron.

[53] Caberlotto E (2011). Usher type 1g protein sans is a critical component of the tip-link complex, a structure controlling actin polymerization in stereocilia. Proc. Natl Acad. Sci. USA.

[54] Lohani S (2022). Spatiotemporally heterogeneous coordination of cholinergic and neocortical activity. Nat. Neurosci..

[55] Mishne, G., Coifman, R. R., Lavzin, M. & Schiller, J. Automated cellular structure extraction in biological images with applications to calcium imaging data. Preprint at bioRxiv 10.1101/313981 (2018).

[56] Pnevmatikakis EA, Giovannucci A (2017). Normcorre: an online algorithm for piecewise rigid motion correction of calcium imaging data. J. Neurosci. Methods.

[57] O’Herron, P., Levy, M., Woodward, J. J. & Kara, P. An unexpected dependence of cortical depth in shaping neural responsiveness and selectivityin mouse visual cortex. *eNeuro* **7**, ENEURO.0497-19.2020 (2020). 10.1523/ENEURO.0497-19.2020 PMC7092962 32051142

[58] Leikvoll A, Kara P (2023). High fidelity sensory-evoked responses in neocortex after intravenous injection of genetically encoded calcium sensors. Front. Neurosci..

[59] Sadakane O (2015). Long-term two-photon calcium imaging of neuronal populations with subcellular resolution in adult non-human primates. Cell Rep..

[60] Chang JT, Whitney D, Fitzpatrick D (2020). Experience-dependent reorganization drives development of a binocularly unified cortical representation of orientation. Neuron.

[61] Lee K-S, Huang X, Fitzpatrick D (2016). Topology of on and off inputs in visual cortex enables an invariant columnar architecture. Nature.

[62] Edelstein A, Amodaj N, Hoover K, Vale R, Stuurman N (2010). Computer control of microscopes using *μ*manager. Curr. Protoc. Mol. Biol..

[63] Peirce JW (2007). Psychopy—psychophysics software in python. J. Neurosci. Methods.

[64] Chang JT, Fitzpatrick D (2022). Development of visual response selectivity in cortical gabaergic interneurons. Nat. Commun..

[65] Lempel AA, Nielsen KJ (2019). Ferrets as a model for higher-level visual motion processing. Curr. Biol..

