# Supplementary material for: The Cousa objective: a long-working distance air objective for multiphoton imaging in vivo
Source: Nat Methods. 2023 Dec 21;21(1):132–41. doi: 10.1038/s41592-023-02098-1 (PMC10776402; doi:10.1038/s41592-023-02098-1)
Supplement: Supplementary file 1 — Reporting Summary [file 41592_2023_2098_MOESM1_ESM.pdf]

nature portfolio

Corresponding author(s): Spencer Smith

Last updated by author(s): Sep 23, 2023

Reporting Summary

Nature Portfolio wishes to improve the reproducibility of the work that we publish. This form provides structure for consistency and transparency in reporting. For further information on Nature Portfolio policies, see our Editorial Policies and the Editorial Policy Checklist.

Statistics

For all statistical analyses, confirm that the following items are present in the figure legend, table legend, main text, or Methods section.

n/a	Confirmed	
☐	☒	The exact sample size (*n*) for each experimental group/condition, given as a discrete number and unit of measurement
☐	☒	A statement on whether measurements were taken from distinct samples or whether the same sample was measured repeatedly
☒	☐	The statistical test(s) used AND whether they are one- or two-sided<br>*Only common tests should be described solely by name; describe more complex techniques in the Methods section.*
☒	☐	A description of all covariates tested
☒	☐	A description of any assumptions or corrections, such as tests of normality and adjustment for multiple comparisons
☐	☒	A full description of the statistical parameters including central tendency (e.g. means) or other basic estimates (e.g. regression coefficient) AND variation (e.g. standard deviation) or associated estimates of uncertainty (e.g. confidence intervals)
☒	☐	For null hypothesis testing, the test statistic (e.g. *F*, *t*, *r*) with confidence intervals, effect sizes, degrees of freedom and *P* value noted<br>*Give P values as exact values whenever suitable.*
☒	☐	For Bayesian analysis, information on the choice of priors and Markov chain Monte Carlo settings
☒	☐	For hierarchical and complex designs, identification of the appropriate level for tests and full reporting of outcomes
☒	☐	Estimates of effect sizes (e.g. Cohen's *d*, Pearson's *r*), indicating how they were calculated

Our web collection on statistics for biologists contains articles on many of the points above.

Software and code

Policy information about availability of computer code

Data collection	Figure 2e, 3a, 3b: ScanImage 2021 (MBF Bioscience).<br>Figure 3c: Commercial softwares (ScanImage 2021, MBF Bioscience) from Zeiss Axiozoom epifluorescence imaging system and Sutter Instruments two-photon imaging system<br>Figure 3d: Commercial Software (PrairieView) from Ultima two-photon microscope (Bruker)<br>Figure 3e: Commercial Software (PrairieView) from Ultima two-photon microscope (Bruker)<br>Figure 4a: ScanImage 2021 (MBF Bioscience)<br>Figure 4b: Commercial software and two-photon microscope from Neurolabware.<br>Figure 4c, 4d: ScanImage 2021 (MBF Bioscience), PsychoPy (v1.85), Spike2 (v7.11b, CED; Cambridge UK).<br>Figure 4e: Commercial Software (PrairieView) from Ultima two-photon microscope (Bruker)
Data analysis	Optical simulations were made using Zemax OpticStudio (v22.2.1). All analyses of the data appearing in the manuscript were performed including Matlab 2019 or newer versions, Suite2p, ImageJ (v1.53f51), Python, Cell Magic Wand (v1.0), and NoRMCorre. Detailed analysis methods were described in the "Methods" section. Any further information needed for replication can be provided upon request.

For manuscripts utilizing custom algorithms or software that are central to the research but not yet described in published literature, software must be made available to editors and reviewers. We strongly encourage code deposition in a community repository (e.g. GitHub). See the Nature Portfolio guidelines for submitting code & software for further information.

Data

Policy information about availability of data

All manuscripts must include a data availability statement. This statement should provide the following information, where applicable:

- Accession codes, unique identifiers, or web links for publicly available datasets
- A description of any restrictions on data availability
- For clinical datasets or third party data, please ensure that the statement adheres to our policy

The lens layout and prescription are fully accessible in Fig. 1. The datasets generated during and/or analyzed during the current study are available from the corresponding author on reasonable request.

Human research participants

Policy information about studies involving human research participants and Sex and Gender in Research.

Reporting on sex and gender	N/A
Population characteristics	N/A
Recruitment	N/A
Ethics oversight	N/A

Note that full information on the approval of the study protocol must also be provided in the manuscript.

Field-specific reporting

Please select the one below that is the best fit for your research. If you are not sure, read the appropriate sections before making your selection.

☒ Life sciences ☐ Behavioural & social sciences ☐ Ecological, evolutionary & environmental sciences

For a reference copy of the document with all sections, see nature.com/documents/nr-reporting-summary-flat.pdf

Life sciences study design

All studies must disclose on these points even when the disclosure is negative.

Sample size	The purpose of this study is to demonstrate the optical performance of a new objective, rather than drawing biological conclusions. Images were acquired from 5 different animal species. Ten mice, one marmoset, one pig, two ferrets, and one tree threw were used, and sufficient to show the objective's capability, while also considering the ethical use and treatment of the experimental animals.
Data exclusions	No data was excluded.
Replication	To determine resolution, 5 beads were imaged at each location in the field of view (Fig. 1c). As this study is to demonstrate the optical performance of a new objective, rather than drawing biological conclusions, replicate experiments were not performed.
Randomization	As this study is to demonstrate the optical performance of a new objective, rather than drawing biological conclusions, randomization does not apply to this study.
Blinding	No blinding was used for this study, as blinding is not required for this study.

Reporting for specific materials, systems and methods

We require information from authors about some types of materials, experimental systems and methods used in many studies. Here, indicate whether each material, system or method listed is relevant to your study. If you are not sure if a list item applies to your research, read the appropriate section before selecting a response.

Materials & experimental systems

n/a	Involved in the study
☒	☐ Antibodies
☒	☐ Eukaryotic cell lines
☒	☐ Palaeontology and archaeology
☐	☒ Animals and other organisms
☒	☐ Clinical data
☒	☐ Dual use research of concern

Methods

n/a	Involved in the study
☒	☐ ChIP-seq
☒	☐ Flow cytometry
☒	☐ MRI-based neuroimaging

Animals and other research organisms

Policy information about studies involving animals; ARRIVE guidelines recommended for reporting animal research, and Sex and Gender in Research

Laboratory animals

Figure 2e, 3a: 2 female GCaMP6s transgenic mice (>5 months) generated by triple crossing of TITL-GCaMP6s mice, Emx1-Cre mice (Jackson Labs stock #005628) and ROSA:LNL:tTA mice (Jackson Labs stock #011008). TITL-GCaMP6s mice were kindly provided by Allen institute.
Figure 3b: Adult (> 8 weeks) C57Bl/6 mice of both sexes (Jackson Labs) were used.
Figure 3c: A c57/Bl6 mouse was used for dual imaging.
Figure 3d: A four-week old male mouse from Ai14tdTomato (JAX: 007908) x Myosin15Cre breeding was used for cochlear hair cell imaging.
Figure 3e: Two C57BL/6J mice were used.
Figure 4a: One four-year-old marmoset participated in this study.
Figure 4b: The experiment was performed in an adult male ferret (Mustela putoris furo; 120 days old, 1.2 kg body weight).
Figure 4c: A juvenile female ferret (P42) was used from Marshal Farms.
Figure 4d: A adult male tree shrew (P155) was used.
Figure 4e: A 6-month-old female Yucatan minipig was obtained from Premier BioSource, CA.

Wild animals

No wild animals were used in this study.

Reporting on sex

This study is to demonstrate the performance of imaging performance, and thus sex of animals was not considered.

Field-collected samples

No field-collected samples were used in this study.

Ethics oversight

Figure 2e, 3a, 3b: All procedures involving living animals for these figures were carried out in accordance with the guidelines and regulations of the US Department of Health and Human Services and approved by the Institutional Animal Care and Use Committee at University of California, Santa Barbara.
Figure 3c: All animal handling and experiments were performed according to the ethical guidelines of the Institutional Animal Care and Use Committee of the Yale University School of Medicine.
Figure 3d: Animal studies were carried out according to the protocols approved by the Institutional Animal Care and Use Committee at Stanford University (APLAC-14345).
Figure 3e: All animal procedures were approved by the Institutional Animal Care and Use Committee at the University of Minnesota.
Figure 4a: All experimental procedures were approved by The Rockefeller University Institutional Animal Care and Use Committee and were performed in accordance with guidelines from the U.S. National Institute of Health.
Figure 4b: All procedures adhered to the guidelines of the National Institute of Health and were approved by the Animal Care and 579 Use Committee at Johns Hopkins University.
Figure 4c, 4d: All experimental procedures were approved by the Max Planck Florida Institute for Neuroscience Institutional Animal Care and Use committee and were performed in accordance with guidelines from the U.S. National Institute of Health.
Figure 4e: The study was conducted with approval by the Administrative Panel for Laboratory Animal Care at Stanford University and in accordance with the Guide for the Care and Use of Laboratory Animals at an AAALAC-accredited facility.

Note that full information on the approval of the study protocol must also be provided in the manuscript.
